# Copper(I)-Catalyzed Formal [4 + 2] Cyclocondensation of *ortho*-Hydroxybenzyl Alcohol, Aromatic Terminal Alkynes, and Sulfonyl Azides: An Alternative Approach to 2-Sulfonyliminocoumarins

**DOI:** 10.3390/molecules29143426

**Published:** 2024-07-22

**Authors:** Dost Muhammad Khan, Jiaying Lv, Ruimao Hua

**Affiliations:** Key Laboratory of Organic Optoelectronics & Molecular Engineering of Ministry of Education, Department of Chemistry, Tsinghua University, Beijing 100084, China

**Keywords:** alkyne, copper-catalyzed, *ortho*-hydroxybenzyl alcohol, sulfonyliminocoumarins

## Abstract

In this paper, an alternative and efficient copper(I)-catalyzed synthesis of 2-sulfonyliminocoumarins is developed through a three-component reaction of *ortho*-hydroxybenzyl alcohol, alkynes, and *p*-toluenesulfonyl azide. The proposed route for access to the 2-iminocoumarin ring involves a [4 + 2] hetero-Diels-Alder reaction between *ortho*-quinone methide and ketenimine intermediates generated in situ.

## 1. Introduction

**Scheme 1:**
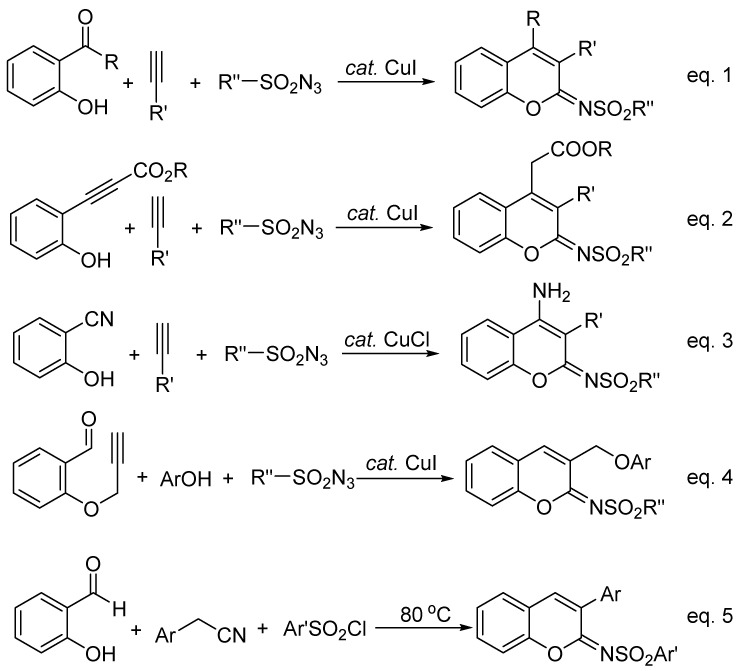
Recent reports on the synthesis of 2-sulfonyliminocoumarins using phenols.

Coumarin-containing compounds are important oxygen-heterocycles that possess notable pharmacological and biological activities [1,2,3,4]. The synthesis, transformation, and biological activity of 2-iminocoumarins are of significant interest in synthetic chemistry and medicinal chemistry [5,6,7,8]. In addition, the *N*-sulfonylimino group is a key motif in organic transformations [9,10,11]; thus, the synthesis of 2-sulfonyliminocoumarins has been well-developed recently (Scheme 1). This includes copper(I)-catalyzed three-component cyclocoupling reactions of alkyne, sulfonyl azide, and *ortho*-functionalized phenols, such as salicylaldehydes (Equation (1)) [12,13], *ortho*-ethynylphenols (Equation (2)) [14], and *ortho*-hydroxybenzonitriles (Equation (3)) [15]; copper(I)-catalyzed cyclocoupling reactions of ynal, phenol, and sulfonyl azide (Equation (4)) [16]; as well as metal catalyst-free cyclocoupling reactions of salicylaldehydes, arylacetonitriles, and arylsulfonyl chlorides in 2-methyl-THF (tetrahydrofuran) solvent (Equation (5)) [17]. Very recently, a Rh(I)/Ag(I)-catalyzed reaction of aryl thiocarbamates, internal alkynes, and sulfonamides was also reported to efficiently afford 2-sulfonyliminocoumarins [18].

**Scheme 2:**
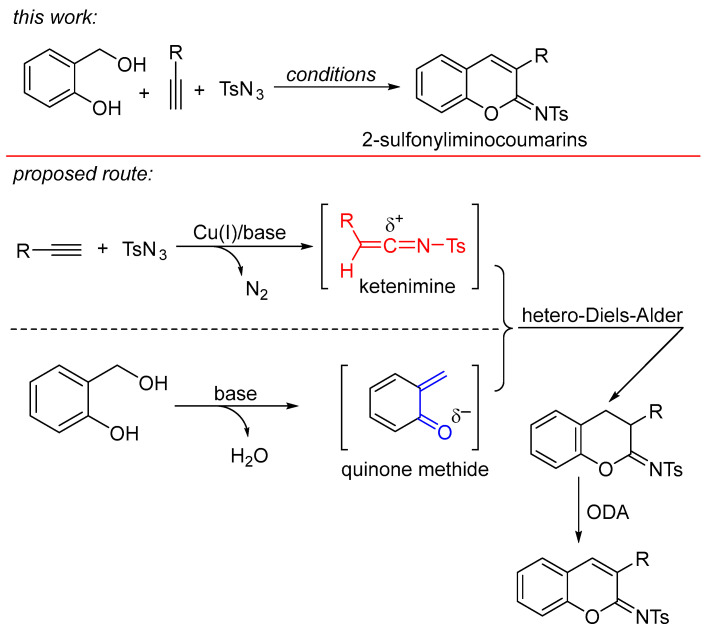
Designed hetero-Diels-Alder reaction of *ortho*-quinone methide with ketenimine for access to 2-sulfonyliminocoumarins.

In a continuation of our studies on the synthesis of oxygen-heterocyclic compounds [19,20,21,22] and the development of alkyne annulations [23], we are interested in establishing an alternative synthetic procedure for the formation of 2-sulfonyliminocoumarins. This involves a three-component cyclocoupling reaction of alkyne, sulfonyl azide, and *ortho*-hydroxybenzyl alcohol, which are cheaper and more stable substrates compared with those used in known procedures. As depicted in Scheme 2, the new designed procedure for accessing 2-sulfonyliminocoumarins involves the formation of two key intermediates: *ortho*-quinone methide [24,25,26] and ketenimine [12,27,28,29], followed by their regioselective [4 + 2] hetero-Diels-Alder reaction and oxidative dehydrogenative aromatization (ODA). 

## 2. Results and Discussion

Based on the above-mentioned formation of ketenimine in the presence of copper (I) salts [12,27,28,29], we examined reactions using various copper (I) salts as catalysts and simple bases in different solvents under an air atmosphere. As summarized in Table 1, in THF solvent with triethylamine (TEA) as the base, CuCl, CuBr, and CuI showed a similar and effective catalytic activity, catalyzing cyclocondensation of 2-(hydroxymethyl)phenol (**1**), phenyl acetylene (**2a**), and *p*-toluenesulfonyl azide (**3**) at 80 °C, affording the desired product **4a** in 65%, 70%, and 65% isolated yields, respectively (entries 1–3). The structure of **4a** was confirmed unambiguously through X-ray diffraction studies [30 and see Supplementary Materials]. When 1,8-diazabicyclo[5.4.0]undec-7-ene (DBU) and K_2_CO_3_ were used as bases, the **4a** yield decreased greatly (entries 4–5). The screening of solvents using TEA showed that 1,2-dichloroethane (DCE) was a better solvent for CuI-catalyzed formation of **4a** (entry 6). DCE was the best solvent when compared with THF, dioxane, DMF (N,N-dimethylformamide), and toluene, with the use of CuTC (copper(I)-thiophene-2-carboxylate) as catalyst to afford **4a** in 82% yield (entries 7–11). When 5.0 mol% of CuTC was used, the yield of **4a** decreased to 51% (entry 12), and in the absence of a catalyst, no desired product formed at all (entry 13). In addition, under a nitrogen atmosphere, the yield of **4a** decreased to 11%.

Then, we examined the reactions of 2-(hydroxymethyl)phenol (**1**) and *p*-toluenesulfonyl azide (**3**) with various aromatic terminal alkynes under the optimized reaction condition (indicated in entry 11 of Table 1) to explore the functional group tolerance. As shown in Table 2, compared with **2a**, 1-naphthyl acetylene (**2b**) and 2-naphthyl acetylene (**2c**) underwent the cyclocondensation to provide slightly lower yields of **4b** and **4c**. Both electron-rich and electron-poor aromatic terminal alkynes showed a good reactivity, resulting in yields of 62–81% for the corresponding products, and the reactions of the electron-rich aromatic terminal alkynes resulted in improved yields for the products. For example, the reactions of aromatic terminal alkynes bearing electron-donating groups, such as Me (**2d**–**2f**), Et (**2g**), *n*-Pr (**2h**), *t*-Bu (**2i**), and MeO (**2j**–**2k**), produced the desired products with yields of 65–81% (**4d**–**4k**). Because of the steric hindrance, *ortho*- and *meta*-substituents resulted in a slightly lower yield (**4d** vs. **4e**, **4j** vs. **4k**). The reactions of electron-poor aromatic terminal alkynes with F (**2l**–**2m**), Cl (**2n**), and Br (**2o**) groups afforded the products (**4l**–**4o**) in 62–73% yields, and these products could easily further derivate with the possible activation of the carbon–halogen bond. In addition, heteroaromatic terminal alkynes (**2p**) could be tolerated in this reaction, resulting in a yield of 76% for the desired product (**4p**). Moreover, the reaction of **1**, **2a**, and benzenesulfonyl azide resulted in a yield of 55% for the corresponding product **4q**, while the reaction of **1**, **2a**, and methanesulfonyl azide did not afford the expected product at all. Note that when aliphatic terminal alkynes were used, none of the expected products formed either.

## 3. Materials and Methods

### 3.1. General Methods

Column chromatography was performed with silica gel and analytical thin-layer chromatography (TLC) was performed on 0.2 mm silica-gel-coated glass sheets. All yields refer to isolated yields. Nuclear magnetic resonance (NMR) spectra were recorded on JEOL 400 (Tokyo, Japan) using CDCl_3_ or DMSO-*d*_6_ as solvents at 298 K. ^1^H NMR (400 MHz) chemical shifts (*δ*) referenced the internal standard TMS (*δ* = 0.00 ppm). ^13^C{^1^H} NMR (101 MHz) chemical shifts referenced internal solvent CDCl_3_ (*δ* = 77.16 ppm) or DMSO-*d*_6_ (δ = 39.52 ppm). High-resolution mass spectra (HRMS) with electron spray ionization (ESI) were obtained with a micrOTOF-Q spectrometer (Agilent, California, CA, USA). Single-crystal X-ray diffraction data were obtained using a SuperNova diffractometer (Tokyo, Japan) with Cu K_α_ radiation at a low temperature (173.15 K).

Caution! *p*-Toluenesulfonyl azide (TsN_3_, **3**) can undergo explosive decomposition at 120°. Although it is safe at 80 °C in a sealed vessel under standard reaction conditions, it is potentially explosive. Therefore, all of the reactions were carried out behind a safety shield in a hood.

### 3.2. Typical Experimental Procedure for the Synthesis of 4-Methyl-N-(3-phenyl-2H-chromen-2-ylidene)benzenesulfonamide (**4a**)

A mixture of 2-(hydroxymethyl)phenol (**1**, 124.1 mg, 1.0 mmol), phenyl acetylene (**2a**, 102.0 mg, 1.0 mmol), sulfonyl azide (**3**, 197.0 mg, 1.0 mmol), TEA (102.0 mg, 1.0 mmol), CuTC (19.0 mg, 0.1 mmol), and DCE (4.0 mL) under an air atmosphere in a 25 mL screw-capped thick-walled Pyrex tube was stirred at 80 °C for 18 h in an oil bath. After the reaction mixture was cooled to room temperature, it was poured into a solvent mixture of water (50.0 mL) and ethyl acetate (20.0 mL), and the two phases were then separated. The aqueous layer was extracted with ethyl acetate (3 × 20.0 mL). The combined organic solvent was dried over anhydrous Na_2_SO_4_. After removal of the organic solvent under reduced pressure, the residue was purified by column chromatography on silica gel with petroleum ether/ethyl acetate (gradient mixture ratio from 100:0 to 80:20) as the eluent to afford **4a** as a pale yellow solid (255.2 mg, 0.82 mmol, 82%). The single crystals of **4a** were obtained by slow evaporation of its solution in a mixture solvent of petroleum ether and CH_2_Cl_2_ at room temperature.

### 3.3. Characterization Data of Products

*4-Methyl-N-(3-phenyl-2H-chromen-2-ylidene)benzenesulfonamide* (**4a**) [12]: Pale yellow solid (255.2 mg, 82%). ^1^H NMR (400 MHz, CDCl_3_) δ 7.95 (d, *J* = 8.3 Hz, 2H), 7.71 (s, 1H), 7.62–7.27 (m, 11H), 2.40 (s, 3H). ^13^C NMR (101 MHz, CDCl_3_) δ 157.5, 152.4, 143.1, 139.7, 139.4, 134.5, 132.0, 130.3, 129.3, 129.2, 129.1, 128.4, 128.2, 127.3, 125.8, 119.9, 116.6, 21.6. HRMS (ESI) *m/z*: [M + H]^+^ Calcd for C_22_H_18_NO_3_S 376.1002, found 376.1006.

*4-Methyl-N-(3-(naphthalen-1-yl)-2H-chromen-2-ylidene)benzenesulfonamide* (**4b**): White solid (235.8 mg, 65%). ^1^H NMR (400 MHz, CDCl_3_) δ 7.89 (d, *J* = 8.0 Hz, 2H), 7.75 (s, 1H), 7.71– 7.37 (m, 11H), 7.13 (d, *J* = 8.0 Hz, 2H), 2.34 (s, 3H). ^13^C NMR (101 MHz, CDCl_3_) δ 158.0, 152.9, 142.9, 141.9, 139.2, 133.7, 132.4, 132.3, 131.5, 130.0, 129.8, 129.5, 129.1, 128.6, 128.3, 127.8, 127.1, 126.6, 126.5, 126.1, 125.4, 125.3, 119.6, 116.9, 21.6. HRMS (ESI) *m*/*z*: [M + H]^+^ Calcd for C_26_H_20_NO_3_S 426.1158, found 426.1162.

*4-Methyl-N-(3-(naphthalen-2-yl)-2H-chromen-2-ylidene)benzenesulfonamide* (**4c**): White solid (246.8 mg, 68%). m.p. 208.4–209.7 °C. ^1^H NMR (400 MHz, CDCl_3_) δ 8.04 (s, 1H), 7.93 (d, *J* = 8.2 Hz, 2H), 7.80–7.76 (m, 6H), 7.71 (d, *J* = 8.5 Hz, 1H), 7.60–7.46 (m, 3H), 7.35 (t, *J* = 7.5 Hz, 1H), 7.26–7.26 (d, *J* = 2.6 Hz, 2H), 2.40 (s, 3H). ^13^C NMR (101 MHz, CDCl_3_) δ 157.6, 152.4, 143.5, 143.1, 140.09, 139.2, 133.4, 133.0, 132.0, 129.7, 129.3, 128.6, 128.4, 128.3, 127.77, 127.71, 127.2, 126.9, 126.6, 126.4, 125.9, 119.9, 116.6, 21.5. HRMS (ESI) *m*/*z*: [M + H] ^+^ Calcd for C_26_H_20_NO_3_S 426.1158, found 426.1162.

*4-Methyl-N-(3-(p-tolyl)-2H-chromen-2-ylidene)benzenesulfonamide* (**4d**) [17]: White solid (260.0 mg, 80%). ^1^H NMR (400 MHz, CDCl_3_) δ 7.95 (d, *J* = 8.2 Hz, 2H), 7.65 (s, 1H), 7.53–7.47 (m, 4H), 7.41 (d, *J* = 8.2 Hz, 1H), 7.31–7.26 (m, 3H), 7.16 (d, *J* = 8.0 Hz, 2H), 2.38 (s, 3H), 2.34 (s, 3H). ^13^C NMR (101 MHz, CDCl_3_) δ 157.5, 152.1, 143.0, 139.3, 139.2, 139.0, 131.7, 131.4, 129.9, 129.2, 128.9, 128.2, 127.3, 125.7, 119.8, 116.2, 21.5, 21.3. HRMS (ESI) *m*/*z*: [M + H]^+^ Calcd for C_23_H_20_NO_3_S 390.1158, found 390.1162.

*4-Methyl-N-(3-(o-tolyl)-2H-chromen-2-ylidene)benzenesulfonamide* (**4e**): White solid (232.2 mg, 71%). ^1^H NMR (400 MHz, CDCl_3_) δ 7.86 (d, *J* = 8.2 Hz, 2H), 7.61–7.55 (m, 2H), 7.50–7.47 (m, 2H), 7.33–7.24 (m, 2H), 7.20–7.10 (m, 5H), 2.38 (s, 3H), 2.25 (s, 3H). ^13^C NMR (101 MHz, CDCl_3_) δ 157.3, 152.7, 143.0, 140.8, 139.4, 136.9, 134.5, 132.0, 131.4, 130.3, 129.8, 129.2, 128.9, 128.2, 127.3, 125.9, 125.8, 119.6, 116.7, 21.6, 21.5. HRMS (ESI) *m*/*z*: [M + H]^+^ Calcd for C_23_H_20_NO_3_S 390.1158, found 390.1162.

*4-Methyl-N-(3-(m-tolyl)-2H-chromen-2-ylidene)benzenesulfonamide* (**4f**): White solid (255.0 mg, 78%). m.p. 177.0–179.0 °C ^1^H NMR (400 MHz, CDCl_3_) δ 7.94 (d, *J* = 8.2 Hz, 2H), 7.69 (s, 1H), 7.57–7.45 (m, 2H), 7.45–7.26 (m, 7H), 7.19 (d, *J* = 8.0 Hz, 1H), 2.39 (s, 3H), 2.36 (s, 3H). ^13^C NMR (101 MHz, CDCl_3_) δ 157.5, 152.3, 143.0, 139.5, 137.9, 134.4, 131.9, 130.3, 129.8, 129.28, 129.26, 128.2, 127.2, 126.3, 125.8, 119.9, 116.6, 21.6, 21.5. HRMS (ESI) *m*/*z*: [M + H]^+^ Calcd for C_23_H_20_NO_3_S 390.1158, found 390.1162.

*N-(3-(4-Ethylphenyl)-2H-chromen-2-ylidene)-4-methylbenzenesulfonamide* (**4g**): White solid (255.7 mg, 75%). m.p. 189.0–190.0 °C. ^1^H NMR (400 MHz, CDCl_3_) δ 7.96 (d, *J* = 8.2 Hz, 2H), 7.67 (s, 1H), 7.52–7.49 (m, 4H), 7.42 (d, *J* = 8.2 Hz, 1H), 7.30–7.27 (m, 3H), 7.22 –7.20 (m, 2H), 2.66 (q, *J* = 7.6 Hz, 2H), 2.39 (s, 3H), 1.25 (t, *J* = 7.6, 3H). ^13^C NMR (101 MHz, CDCl_3_) δ 157.6, 152.2, 145.4, 143.1, 139.3, 139.2, 131.8, 131.7, 130.0, 129.2, 129.1, 128.2, 127.8, 127.4, 125.7, 119.9, 116.3, 28.7, 21.6, 15.4. HRMS (ESI) *m*/*z*: [M + H]^+^ Calcd for C_24_H_22_NO_3_S 404.1315, found 404.1319.

*4-Methyl-N-(3-(4-propylphenyl)-2H-chromen-2-ylidene)benzenesulfonamide* (**4h**): White solid (277.2 mg, 78%). m.p. 199.0–201.0 °C. ^1^H NMR (400 MHz, CDCl_3_) δ 7.99–7.94 (m, 2H), 7.68–7.67 (m, 1H), 7.57–7.41 (m, 5H), 7.36–7.26 (m, 3H), 7.23–7.18 (m, 2H), 2.61 (t, *J* = 7.6, 2H), 2.40 (s, 3H), 1.72–1.60 (m, 2H), 0.97 (t, *J* = 7.2, 3H). ^13^C NMR (101 MHz, CDCl_3_) δ 157.6, 152.2, 143.9, 143.1, 139.4, 139.2, 131.8, 130.1, 129.2, 129.0, 128.4, 128.1, 127.4, 125.7, 120.0, 116.4, 37.9, 24.4, 21.6, 13.9. HRMS (ESI) *m*/*z*: [M + H]^+^ Calcd for C_25_H_24_NO_3_S 418.1471, found 418.1475.

*N-(3-(4-(t-Butyl)phenyl)-2H-chromen-2-ylidene)-4-methylbenzenesulfonamide* (**4i**): White solid (298.9 mg, 81%). m.p. 193.2–194.9 °C. ^1^H NMR (400 MHz, CDCl_3_) δ 7.98 (d, *J* = 8.2 Hz, 2H), 7.69 (s, 1H), 7.59–7.49 (m, 4H), 7.42–7.34 (m, 3H), 7.34–7.26 (m, 3H), 2.40 (s, 3H), 1.34 (s, 9H). ^13^C NMR (101 MHz, CDCl_3_) δ 157.6, 152.3, 143.1, 139.38, 139.31, 131.8, 131.5, 130.1, 129.3, 128.8, 128.2, 127.5, 125.8, 125.4, 120.0, 116.4, 34.8, 31.3, 21.6. HRMS (ESI) *m*/*z*: [M + H]^+^ Calcd for C_26_H_26_NO_3_S 432.1628, found 432.1632.

*N-(3-(4-Methoxyphenyl)-2H-chromen-2-ylidene)-4-methylbenzenesulfonamide* (**4j**) [12]: White solid (259.2 mg, 76%). ^1^H NMR (400 MHz, CDCl_3_) δ ^1^H NMR (400 MHz, CDCl_3_) δ 7.96 (d, *J* = 8.3 Hz, 2H), 7.66 (s, 1H), 7.58–7.43 (m, 4H), 7.35–7.27 (m, 4H), 6.92 (d, *J* = 8.3 Hz, 2H), 3.84 (s, 3H), 2.40 (s, 3H). ^13^C NMR (101 MHz, CDCl_3_) δ 160.3, 157.6, 152.1, 143.0, 139.3, 138.5, 131.6, 130.5, 129.7, 129.2, 128.0, 127.3, 126.7, 125.7, 120.0, 116.4, 113.7, 55.4, 21.6. HRMS (ESI) *m*/*z*: [M + H]^+^ Calcd for C_23_H_20_NO_4_S 406.1108, found 406.1112.

*N-(3-(3-Methoxyphenyl)-2H-chromen-2-ylidene)-4-methylbenzenesulfonamide* (**4k**): White solid (222.8 mg, 65%). ^1^H NMR (400 MHz, CDCl_3_) δ 7.95 (d, *J* = 8.2 Hz, 2H), 7.71 (s, 1H), 7.58–7.50 (m, 2H), 7.44 (d, *J* = 8.2 Hz, 1H), 7.35–7.28 (m, 4H), 7.16–7.13 (m, 2H), 6.91– 6.88 (m, 1H), 3.73 (s, 3H), 2.39 (s, 3H). ^13^C NMR (101 MHz, CDCl_3_) δ 159.3, 157.3, 152.2, 143.1, 139.8, 139.4, 135.6, 132.0, 129.7, 129.3, 129.2, 128.3, 127.2, 125.8, 121.4, 119.7, 116.4, 115.0, 114.5, 55.3, 21.5. HRMS (ESI) *m*/*z*: [M + H]^+^ Calcd for C_23_H_20_NO_4_S 406.1108, found 406.1112.

*N-(3-(4-Fluorophenyl)-2H-chromen-2-ylidene)-4-methylbenzenesulfonamide* (**4l**): White solid (240.2 mg, 73%). m.p. 194.2–196.0 °C. ^1^H NMR (400 MHz, CDCl_3_) δ 7.92 (d, *J* = 8.3 Hz, 2H), 7.78 (d, *J* = 8.2 Hz, 1H), 7.67 (s, 1H), 7.60–7.49 (m, 3H), 7.44–7.42 (m, 1H), 7.33 (t, *J* = 7.5 Hz, 1H), 7.27–7.26 (m, 2H), 7.05 (t, *J* = 8.6 Hz, 2H), 2.38 (s, 3H). ^13^C NMR (101 MHz, CDCl_3_) δ 163.1 (d, *J*_C-F_ = 249.1 Hz), 157.4, 152.3, 143.2, 139.6, 139.2, 132.1, 131.1, 130.4, 129.7, 129.3, 128.2, 127.3, 126.5, 125.9, 119.7, 116.5, 115.5, 115.2, 21.6. HRMS (ESI) *m*/*z*: [M + H]^+^ Calcd for C_22_H_17_FNO_3_S 394.0908, found 394.0904.

*N-(3-(2-Fluorophenyl)-2H-chromen-2-ylidene)-4-methylbenzenesulfonamide* (**4m**) [17]: White solid (225.0 mg, 68%). ^1^H NMR (400 MHz, CDCl_3_) δ 7.90–7.75 (m, 3H), 7.70 (s, 1H), 7.59–7.47 (m, 2H), 7.44–7.40 (m, 1H), 7.39–7.30 (m, 2H), 7.25–7.06 (m, 4H), 2.37 (s, 3H). ^13^C NMR (101 MHz, CDCl_3_) δ 159.9 (d, *J*_C-F_ = 249.2 Hz), 157.0, 152.5, 143.3 (d, *J*_C-F_ = 16.9 Hz), 139.2 (d, *J*_C-F_ = 31.2 Hz), 132.4, 131.4, 130.9 (d, *J*_C-F_ = 8.4 Hz), 129.6, 129.2, 128.4, 127.3, 126.4, 125.9, 124.1 (d, *J*_C-F_ = 3.2 Hz), 122.2 (d, *J*_C-F_ = 14.2 Hz), 119.3, 116.5, 115.8, 21.7. HRMS (ESI) *m*/*z*: [M + H]^+^ Calcd for C_22_H_17_FNO_3_S 394.0908, found 394.0912.

*N-(3-(3-Chlorophenyl)-2H-chromen-2-ylidene)-4-methylbenzenesulfonamide* (**4n**): White solid (235.6 mg, 68%). ^1^H NMR (400 MHz, CDCl_3_) δ 7.94 (d, *J* = 8.2 Hz, 2H), 7.72 (s, 1H), 7.62–7.48 (m, 5H), 7.39–7.28 (m, 5H), 2.41 (s, 3H). ^13^C NMR (101 MHz, CDCl_3_) δ 157.1, 152.5, 143.2, 140.2, 139.3, 136.2, 134.2, 132.4, 129.6, 129.4, 129.3, 129.2, 128.9, 128.4, 127.4, 127.2. 126.0, 119.6, 116.7, 21.7. HRMS (ESI) *m*/*z*: [M + H]^+^ Calcd for C_22_H_17_ClNO_3_S 410.0612, found 410.0616.

*N-(3-(3-Bromophenyl)-2H-chromen-2-ylidene)-4-methylbenzenesulfonamide* (**4o**): White solid (242.2 mg, 62%). ^1^H NMR (400 MHz, CDCl_3_) δ 7.95 (d, *J* = 8.2 Hz, 2H), 7.75 (s, 1H), 7.73 (s, 1H), 7.62–7.27 (m, 5H), 7.38 (t, *J* = 7.5 Hz, 1H), 7.34–7.27 (m, 3H), 2.42 (s, 3H). ^13^C NMR (101 MHz, CDCl_3_) δ 157.0, 152.5, 143.2, 140.1, 139.3, 136.4, 132.4, 132.1, 132.0, 129.9, 129.4, 128.7, 128.4, 127.9, 127.2, 126.0, 122.3, 119.6, 116.7, 21.7. HRMS (ESI) *m*/*z*: [M + H]^+^ Calcd for C_22_H_17_BrNO_3_S 456.0088, found 456.0092.

*4-Methyl-N-(3-(thiophen-2-yl)-2H-chromen-2-ylidene)benzenesulfonamide* (**4p**): Pale yellow solid (242.4 mg, 76%). m.p. 181.0–183.0 °C. ^1^H NMR (400 MHz, CDCl_3_) δ 8.02 (d, *J* = 7.6 Hz, 2 H), 7.96–7.63 (s, 1H), 7.69–7.68 (m, 1H), 7.55–7.40 (m, 3H), 7.38–7.28 (m, 4H), 7.06–7.03 (m, 1H), 2.41 (s, 3H). ^13^C NMR (101 MHz, CDCl_3_) δ 155.5, 151.5, 143.3, 139.2, 135.5, 134.9, 131.8, 129.4, 128.9, 128.1, 127.7, 127.4, 127.1, 125.9, 123.1, 119.5, 116.4, 21.7. HRMS (ESI) *m*/*z*: [M + H]^+^ Calcd for C_20_H_16_NO_3_S_2_ 382.0566, found 382.0570.

*N-(3-Phenyl-2H-chromen-2-ylidene)benzenesulfonamide* (**4q**): White solid (201.3 mg, 55%). m.p. 174.0–176.0 °C. ^1^H NMR (400 MHz, DMSO-*d*_6_) δ 8.37 (s, 1H), 8.23 (d, *J* = 6.9 Hz, 1H), 8.07–7.93 (m, 2H), 7.88–7.70 (m, 6H), 7.69–7.51 (m, 5H). ^13^C NMR (100 MHz, DMSO-*d*_6_) δ 157.3, 151.4, 144.1, 141.6, 141.4, 134.3, 132.8, 132.5, 131.7, 129.1, 129.0, 128.9, 128.1, 127.1, 125.5, 119.6, 115.5. HRMS (ESI) *m*/*z*: [M + H]^+^ Calcd for C_21_H_16_NO_3_S 362.0870, found 362.0866.

## 4. Conclusions

In conclusion, the syntheses of 2-sulfonyliminocoumarins with decent yields and high atom utilization are developed via a CuTC-catalyzed three-component cyclocondendation of *ortho*-hydroxybenzyl alcohol, *p*-toluenesulfonyl azide, and various aromatic terminal alkynes in the presence of a base. The proposed way access the 2-iminocoumarin ring involves a [4 + 2] hetero-Diels-Alder reaction between *ortho*-quinone methide and ketenimine intermediate, which have an interesting application in the formation of functionalized oxygen-containing heterocyclic compounds.

## Figures and Tables

**Table 1:** Optimization of the reaction conditions *^a^*.

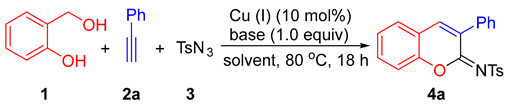				
Entry	Catalyst	Base	Solvent	Yield (%) *^b^*
1	CuCl	TEA	THF	65
2	CuBr	TEA	THF	70
3	CuI	TEA	THF	65
4	CuI	DBU	THF	30
5	CuI	K_2_CO_3_	THF	40
6	CuI	TEA	DCE	78
7	CuTC	TEA	THF	75
8	CuTC	TEA	dioxane	78
9	CuTC	TEA	DMF	58
10	CuTC	TEA	toluene	<5
11	CuTC	TEA	DCE	82
12 *^c^*	CuTC	TEA	DCE	51
13	-	TEA	DCE	-
14 *^d^*	CuTC	TEA	DCE	11

*^a^* Reactions were carried out using 1.0 mmol of **1**, 1.0 mmol of **2a**, 1.0 mmol of **3**, 1.0 mmol of base, and 0.1 mmol of copper (I) salts in 4.0 mL of solvent under an air atmosphere. *^b^* Isolated yields. *^c^* 5.0 mol% of CuTC was used. *^d^* Under a nitrogen atmosphere.

**Table 2:** Scope for the formation of 2-sulfonyliminocoumarins *^a^*.

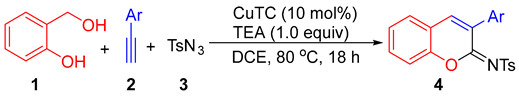		
Ar	Product	Yield (%)
α-naphthyl	**4b**	65
β-naphthyl	**4c**	68
*p*-tolyl	**4d**	80
*o*-tolyl	**4e**	71
*m*-tolyl	**4f**	78
*p*-EtC_6_H_4_	**4g**	75
*p-n*-PrC_6_H_4_	**4h**	78
*p-t*-BuC_6_H_4_	**4i**	81
*p*-MeOC_6_H_4_	**4j**	76
*m*-MeOC_6_H_4_	**4k**	65
*p*-FC_6_H_4_	**4l**	73
*o*-FC_6_H_4_	**4m**	68
*m*-ClC6H4	**4n**	68
*m*-BrC_6_H_4_	**4o**	62
2-thienyl	**4p**	76
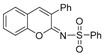	**4q**	55

*^a^* Reactions were carried out using 1.0 mmol of **1**, 1.0 mmol of **2**, 1.0 mmol of **3**, 1.0 mmol of TEA, and 0.1 mmol of CuTC in 4.0 mL of DCE under an air atmosphere.

## Data Availability

Data are contained within the article and Supplementary Materials.

## Supplementary Materials

The following supporting information can be downloaded at: https://www.mdpi.com/article/10.3390/molecules29143426/s1: the copies of NMR charts of products, X-ray structural details of **4a**. References [31,32,33] are cited in the Supplementary Materials.
