# Supplementary material for: Copper(I)-Catalyzed Formal [4 + 2] Cyclocondensation of ortho-Hydroxybenzyl Alcohol, Aromatic Terminal Alkynes, and Sulfonyl Azides: An Alternative Approach to 2-Sulfonyliminocoumarins
Source: Molecules. 2024 Jul 22;29(14):3426. doi: 10.3390/molecules29143426 (PMC11279675; doi:10.3390/molecules29143426)
Supplement: Supplementary file 1 [file molecules-29-03426-s001.zip › molecules-2988972-supplementary.pdf]

Supplementary Materials

Copper(I)-catalyzed Formal [4 + 2] cyclocondensation of *ortho*-Hydroxybenzyl Alcohol with, Aromatic Terminal Alkynes and Sulfonyl Azides: An Alternative Approach to 2-Sulfonyliminocoumarins

Dost Muhammad Khan, Jiaying Lv, Hua Ruimao*

Key Laboratory of Organic Optoelectronics & Molecular Engineering of Ministry of Education, Department of Chemistry, Tsinghua University, Beijing 100084, China

E-mail: ruimao@mail.tsinghua.edu.cn

Contents | Page

1. Copies of NMR Spectra of Products S2

2. X-ray structural details of **4a** S19

1. Copies of NMR Spectra of Products

^{1}H NMR spectrum of compound **4a**

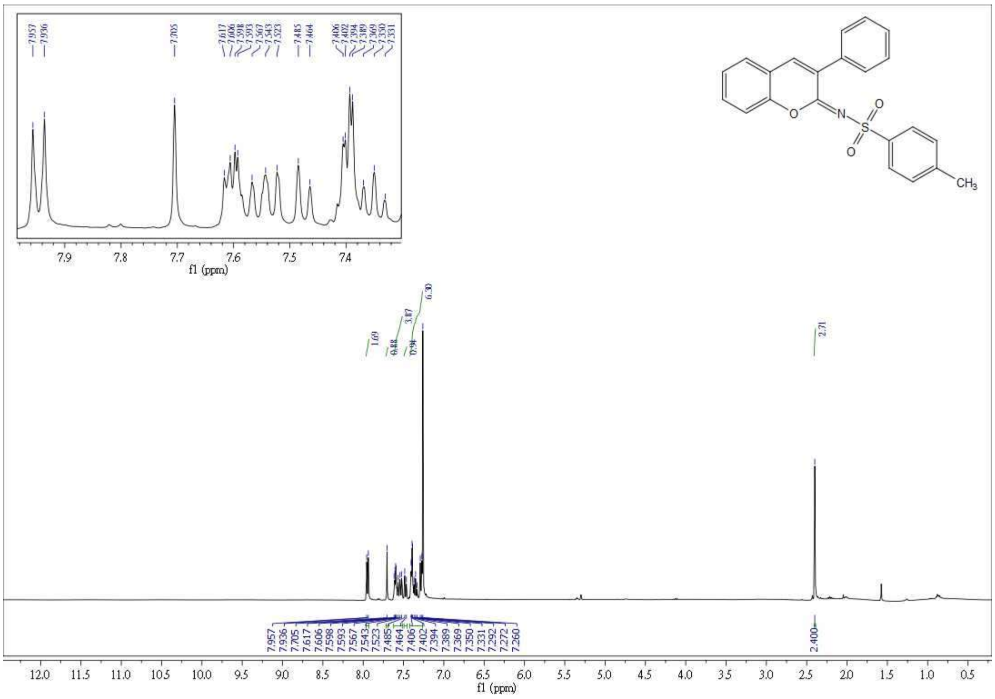

^{13}C NMR spectrum of compound **4a**

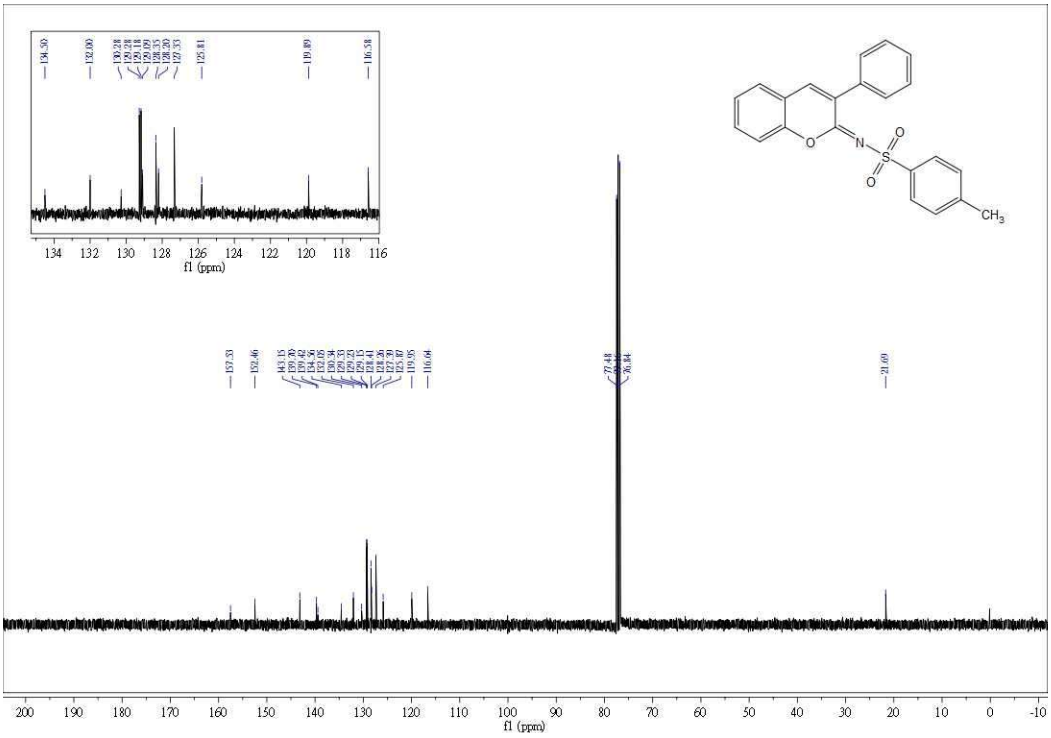

^{1}H NMR spectrum of compound **4b**

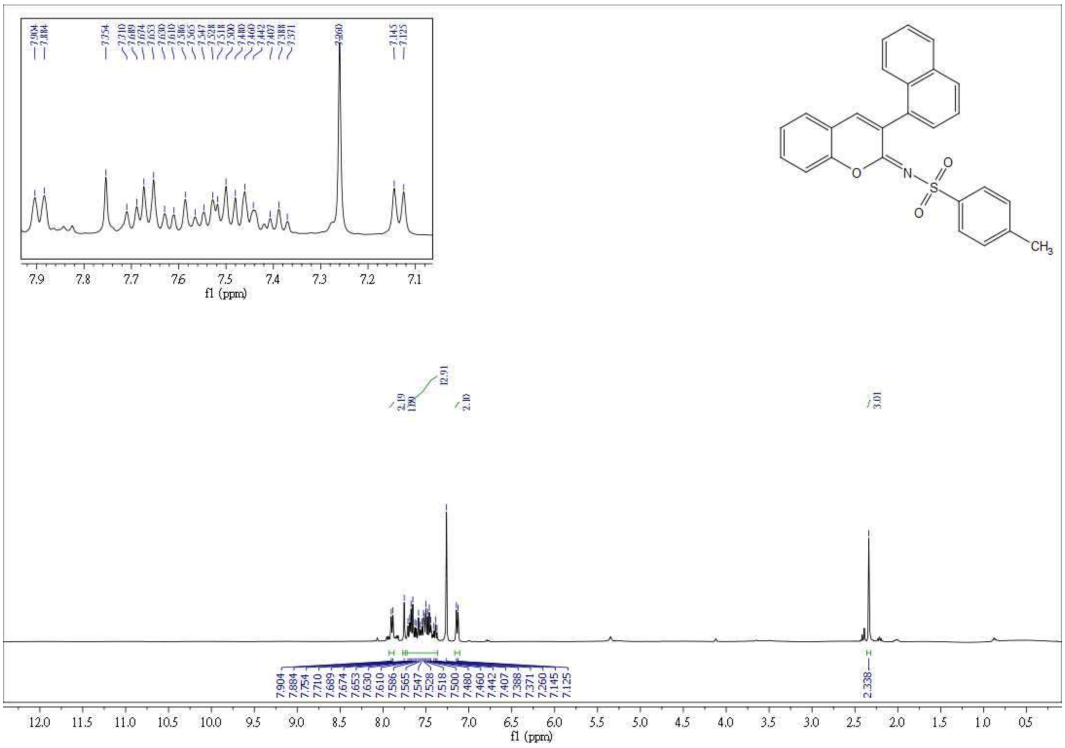

^{13}C NMR spectrum of compound **4b**

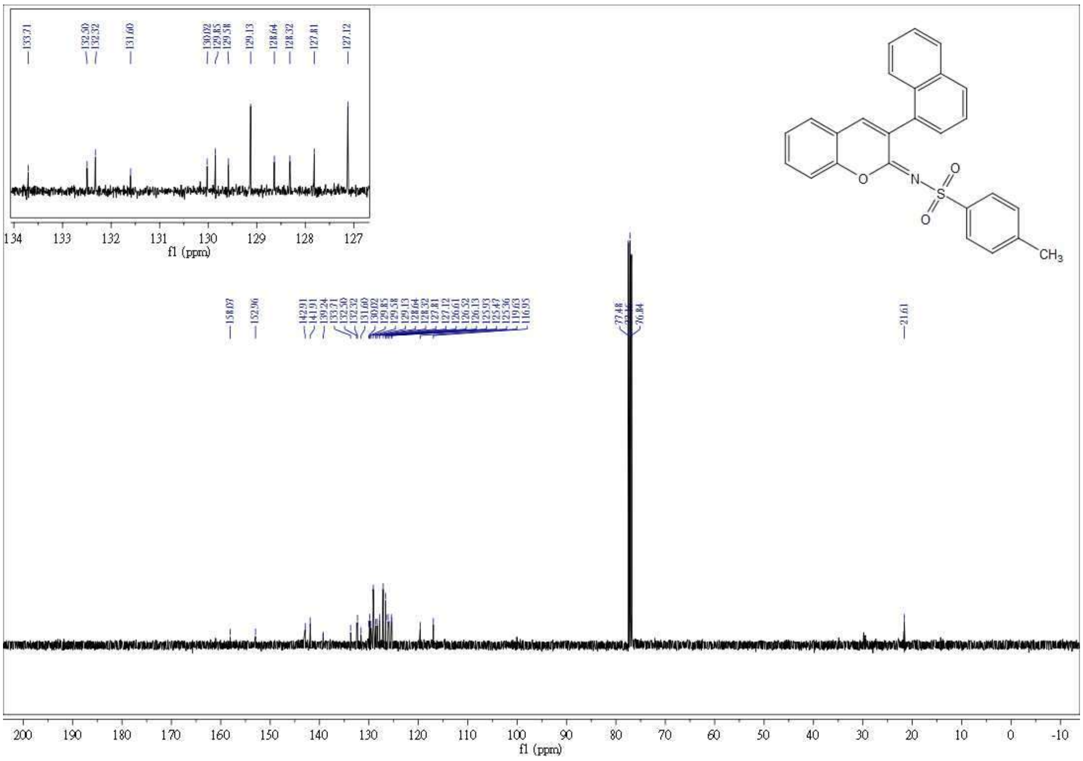

^{1}H NMR spectrum of compound **4c**

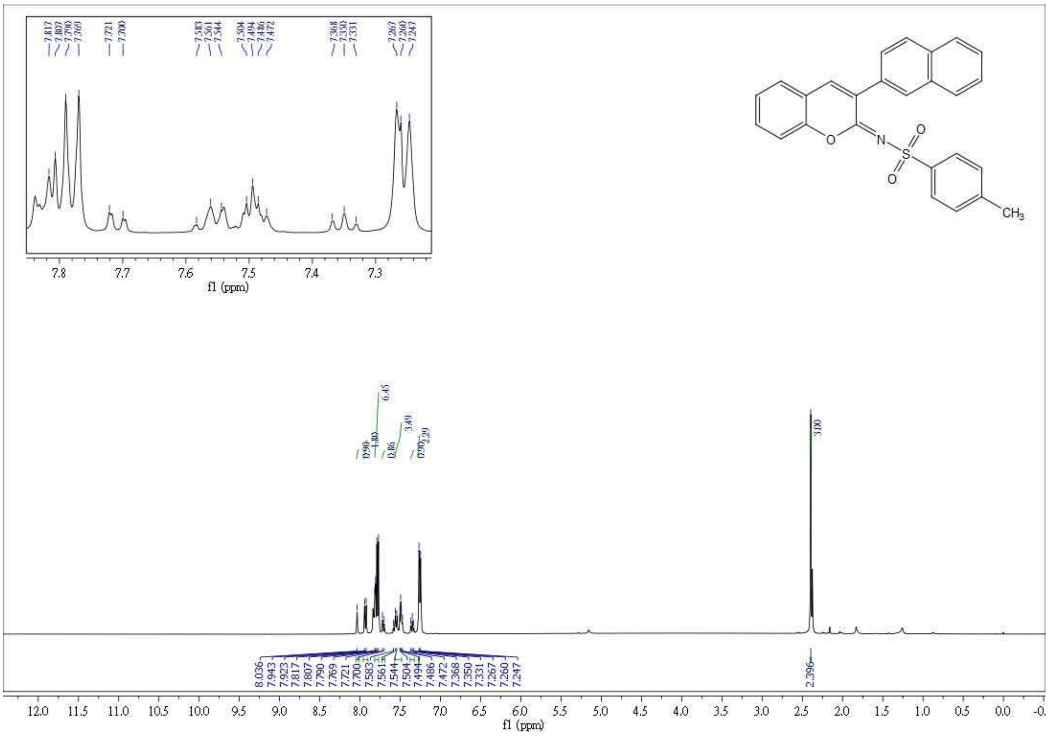

^{13}C NMR spectrum of compound **4c**

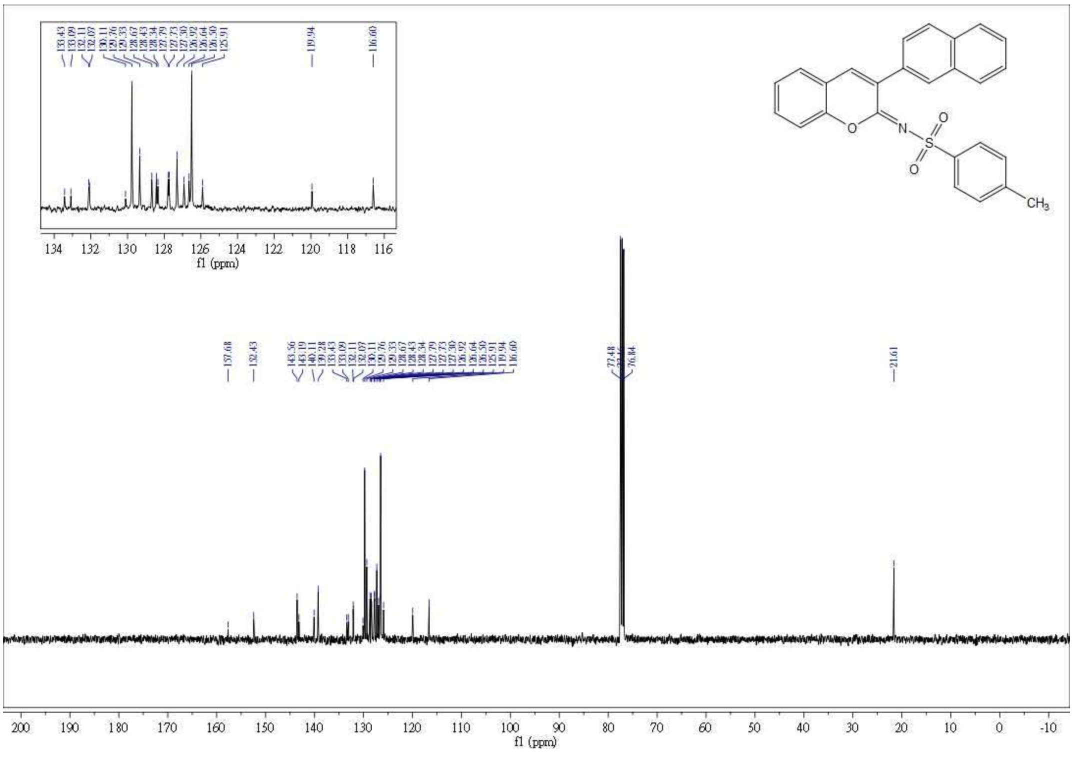

^{1}H NMR spectrum of compound **4d**

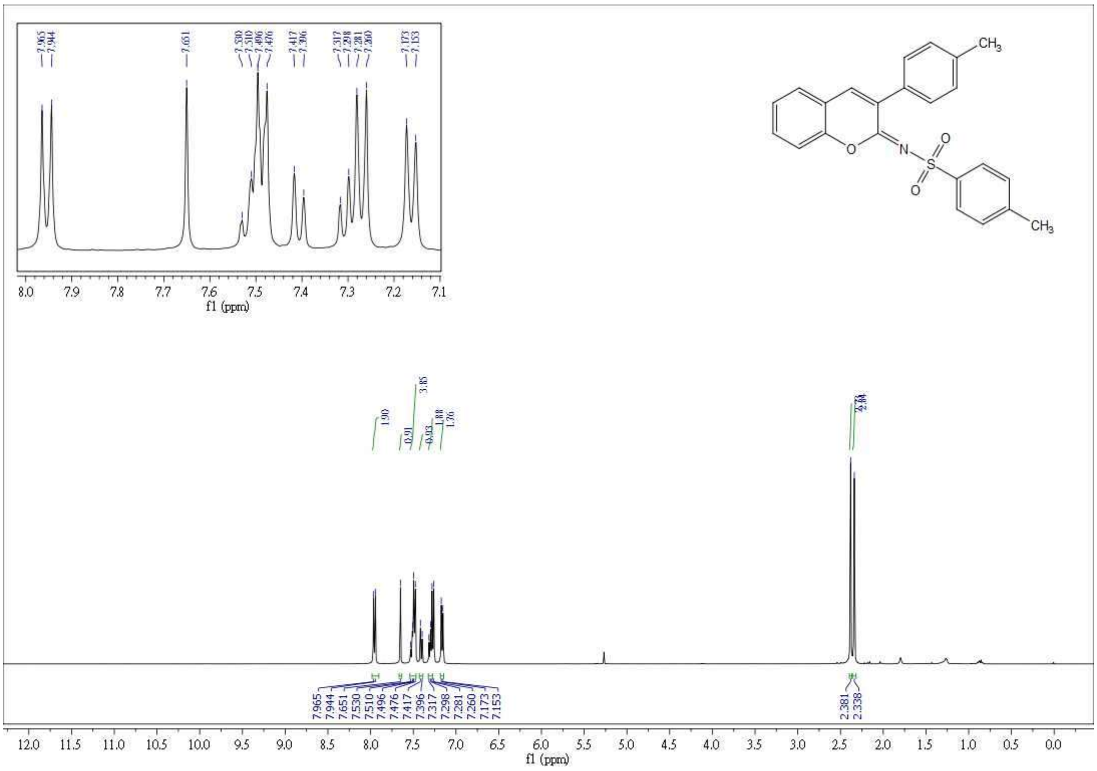

^{13}C NMR spectrum of compound **4d**

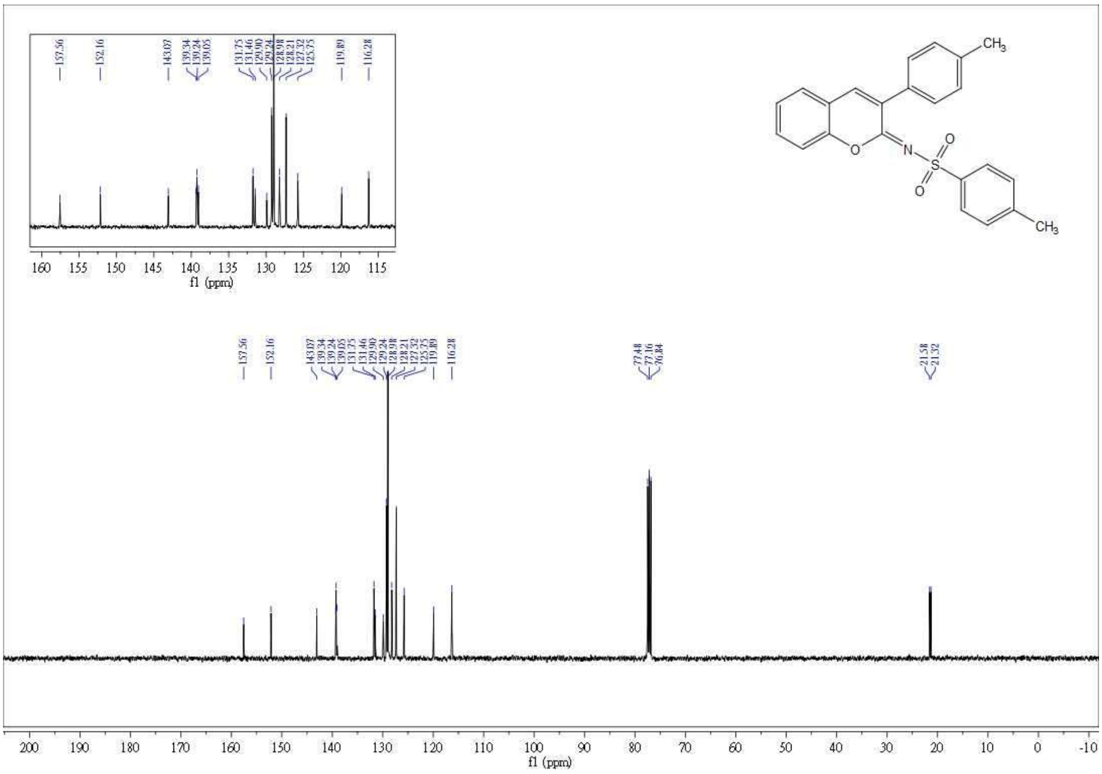

^{1}H NMR spectrum of compound **4e**

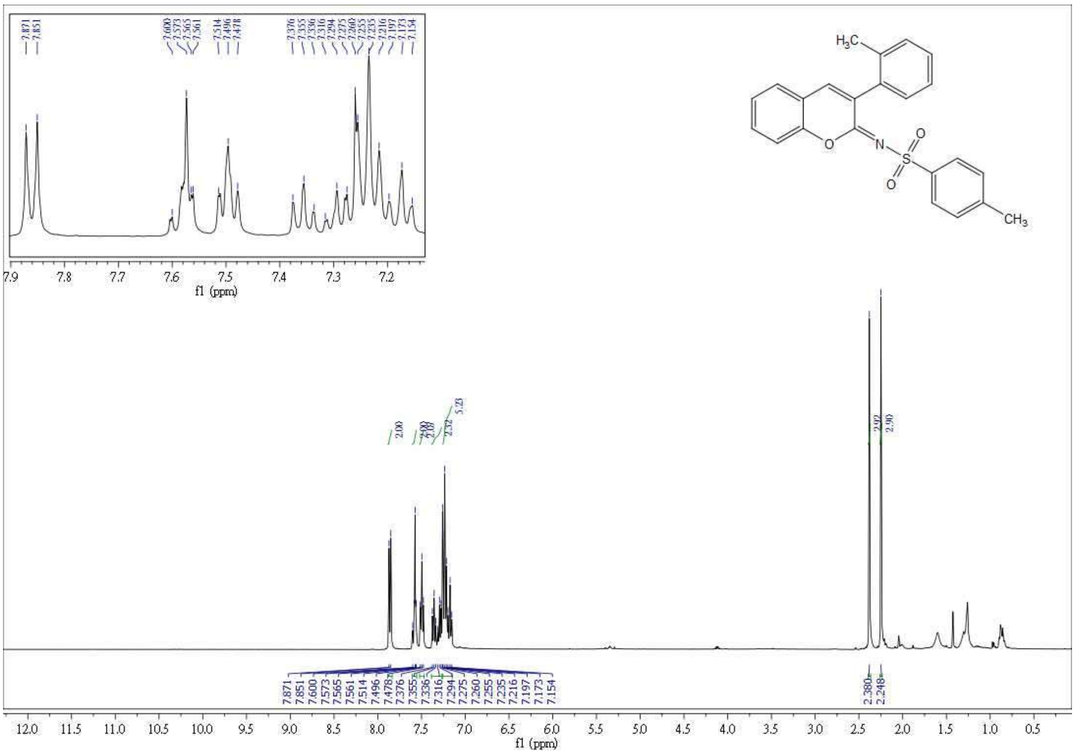

^{13}C NMR spectrum of compound **4e**

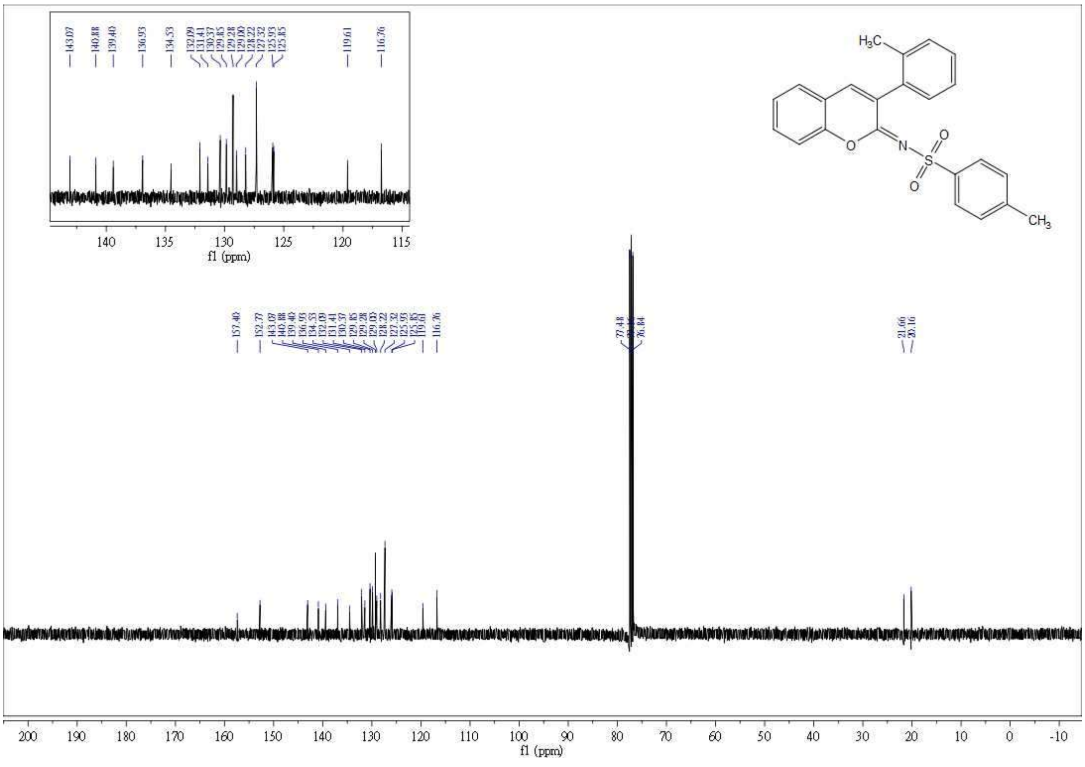

^{1}H NMR spectrum of compound **4f**

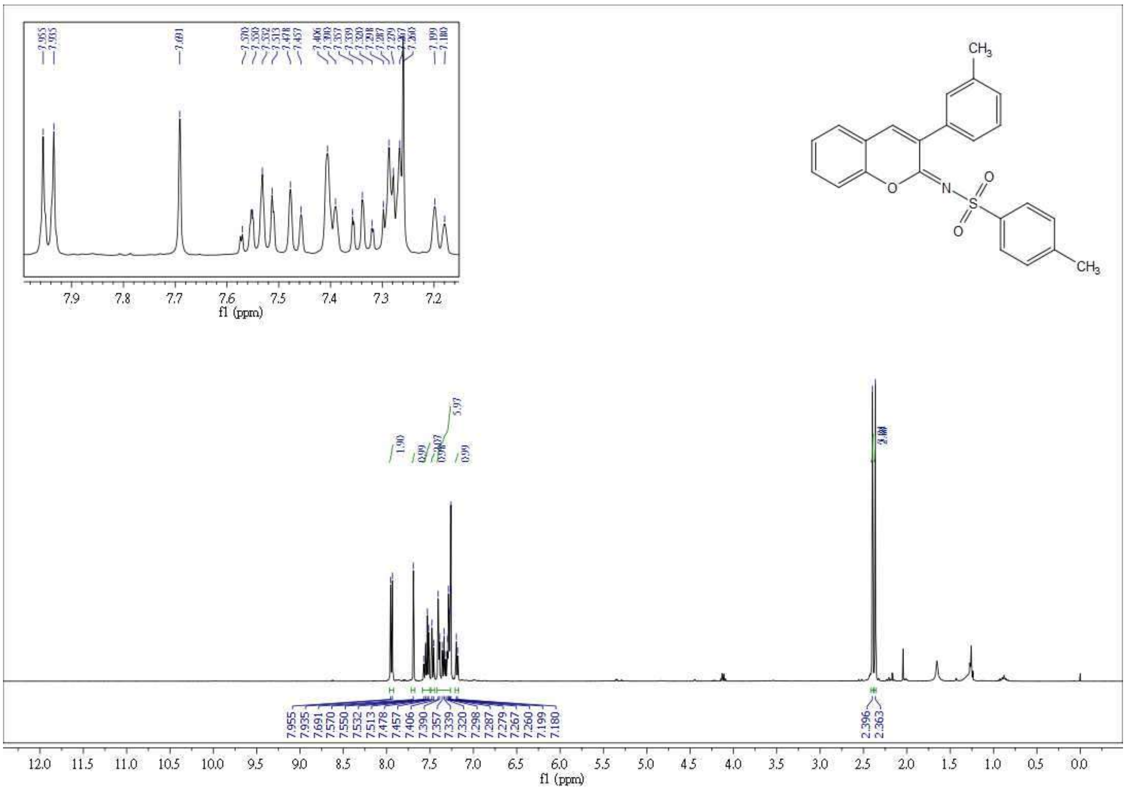

^{13}C NMR spectrum of compound **4f**

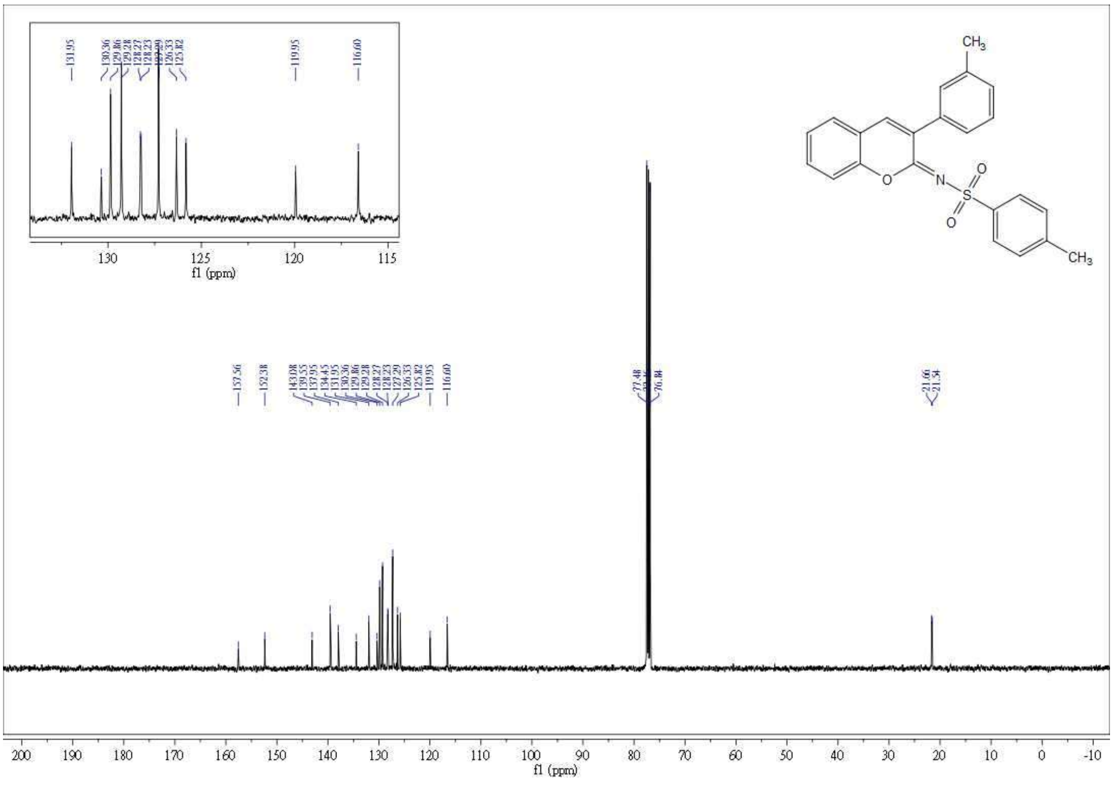

^{1}H NMR spectrum of compound **4g**

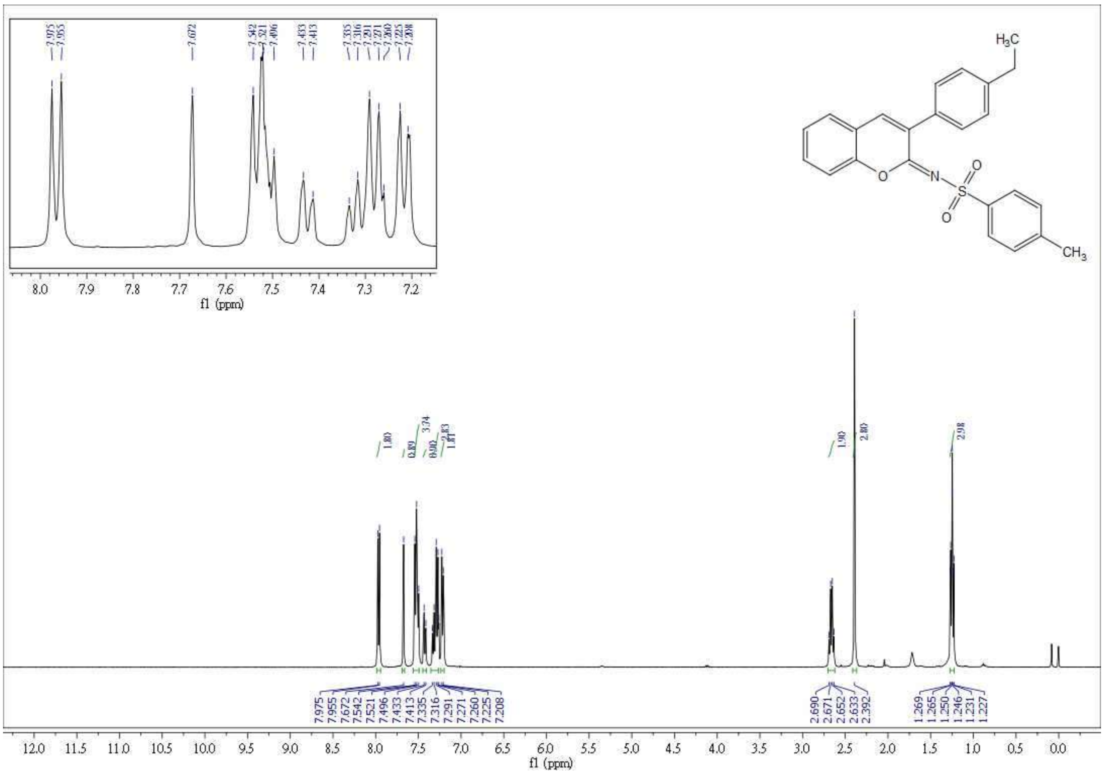

^{13}C NMR spectrum of compound **4g**

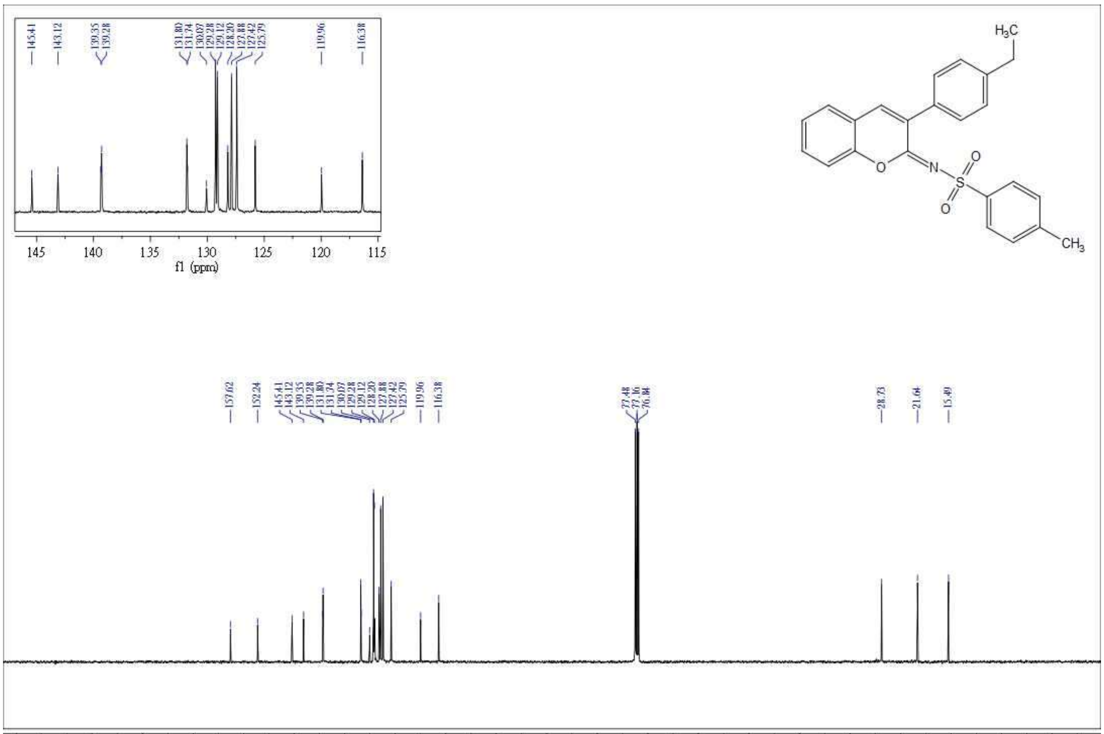

^{1}H NMR spectrum of compound **4h**

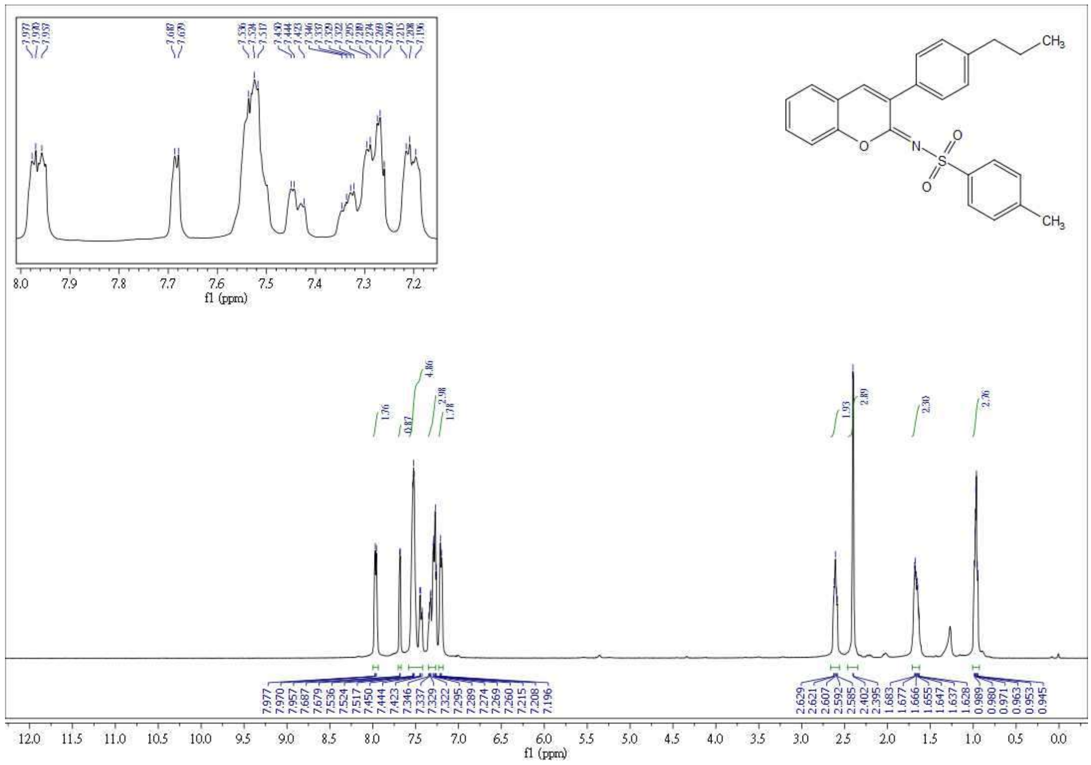

^{13}C NMR spectrum of compound **4h**

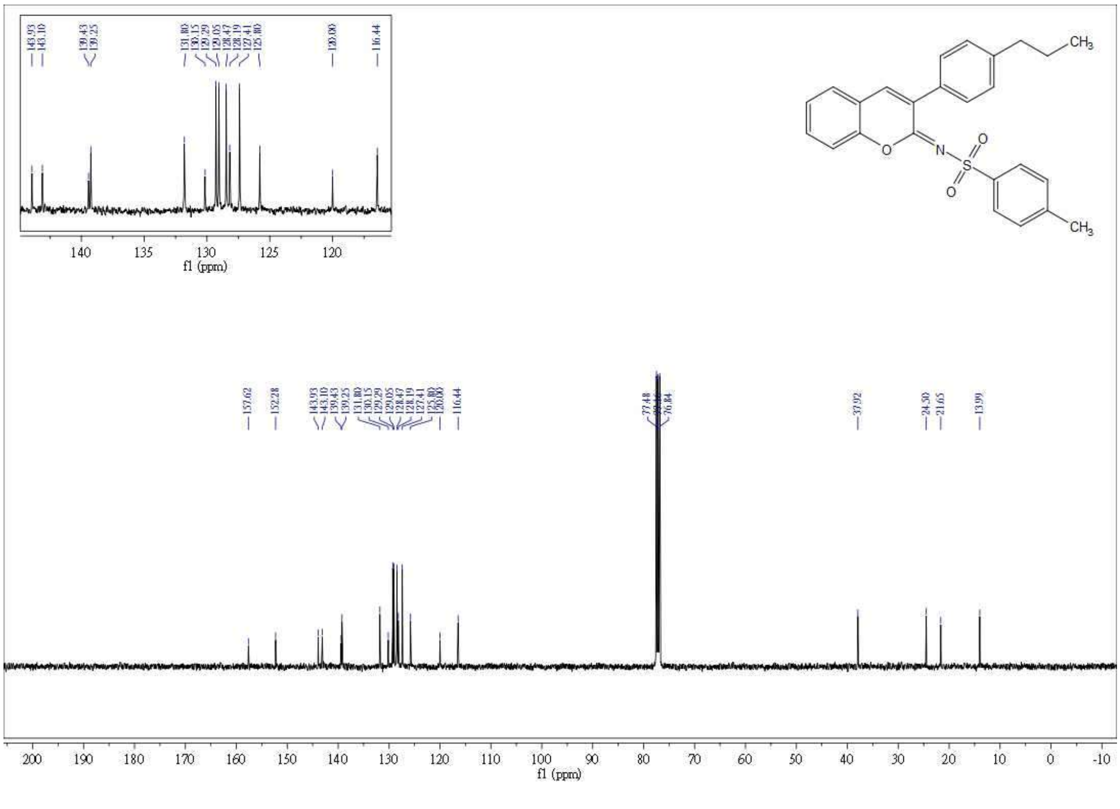

^{1}H NMR spectrum of compound **4i**

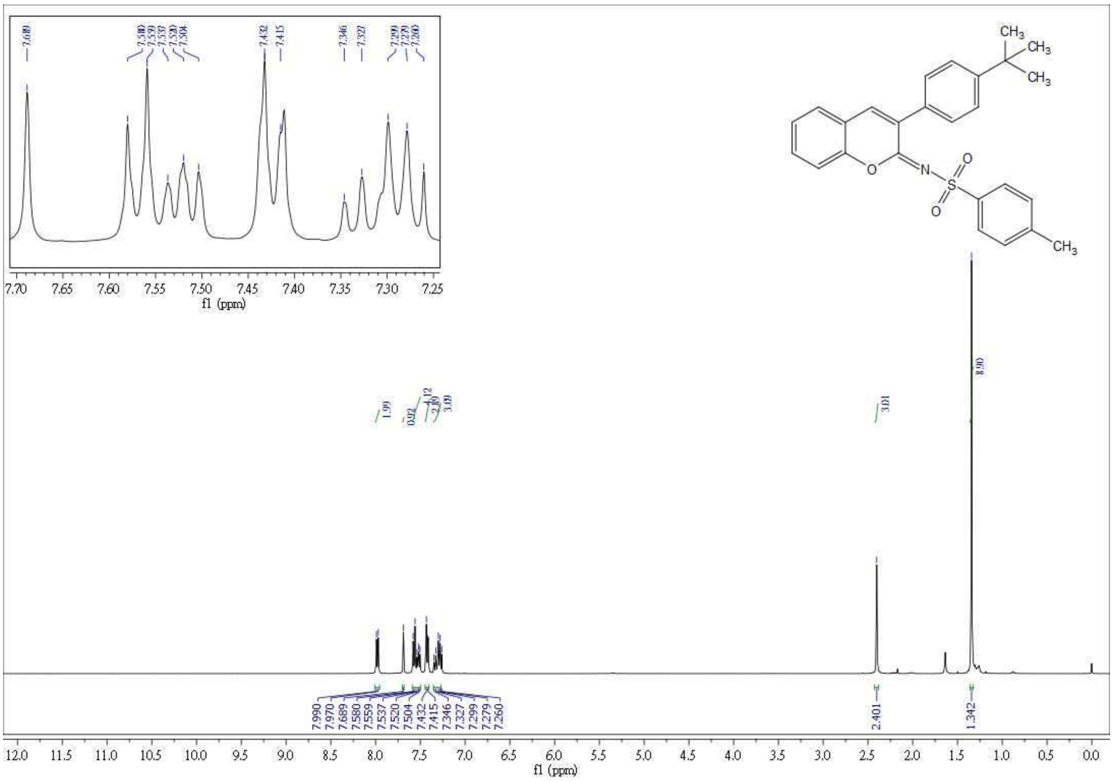

^{13}C NMR spectrum of compound **4i**

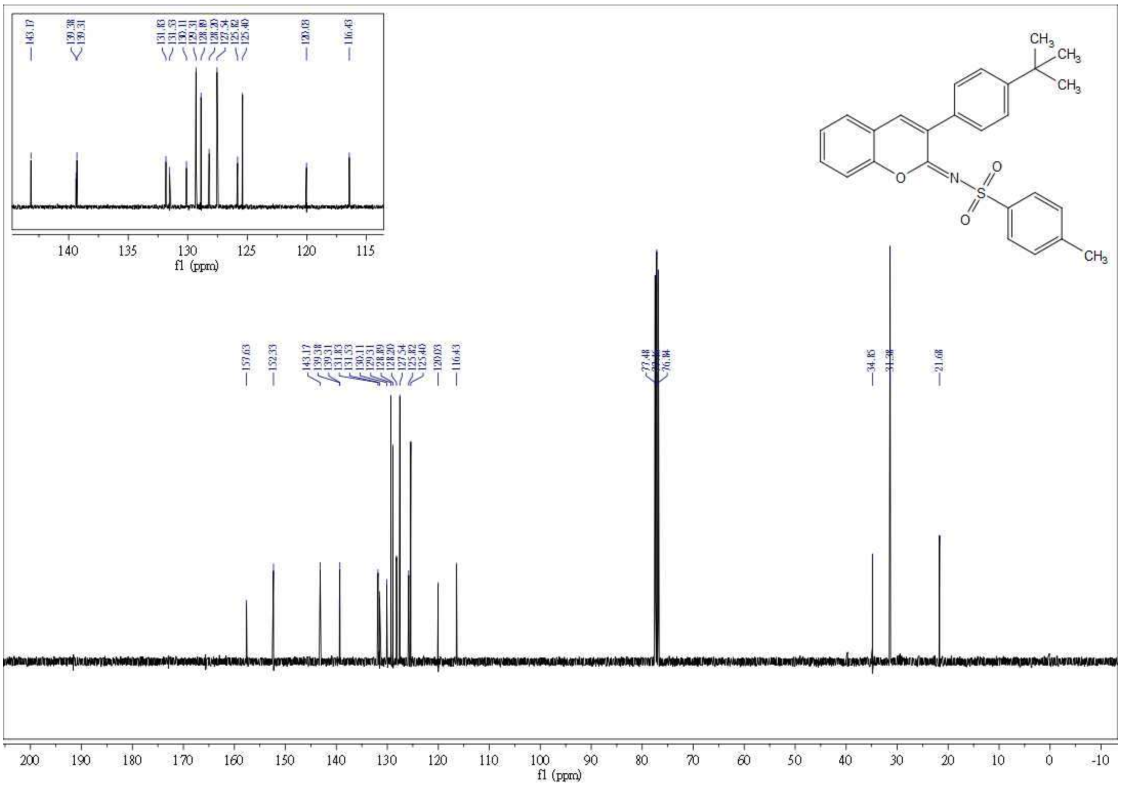

^{1}H NMR spectrum of compound **4j**

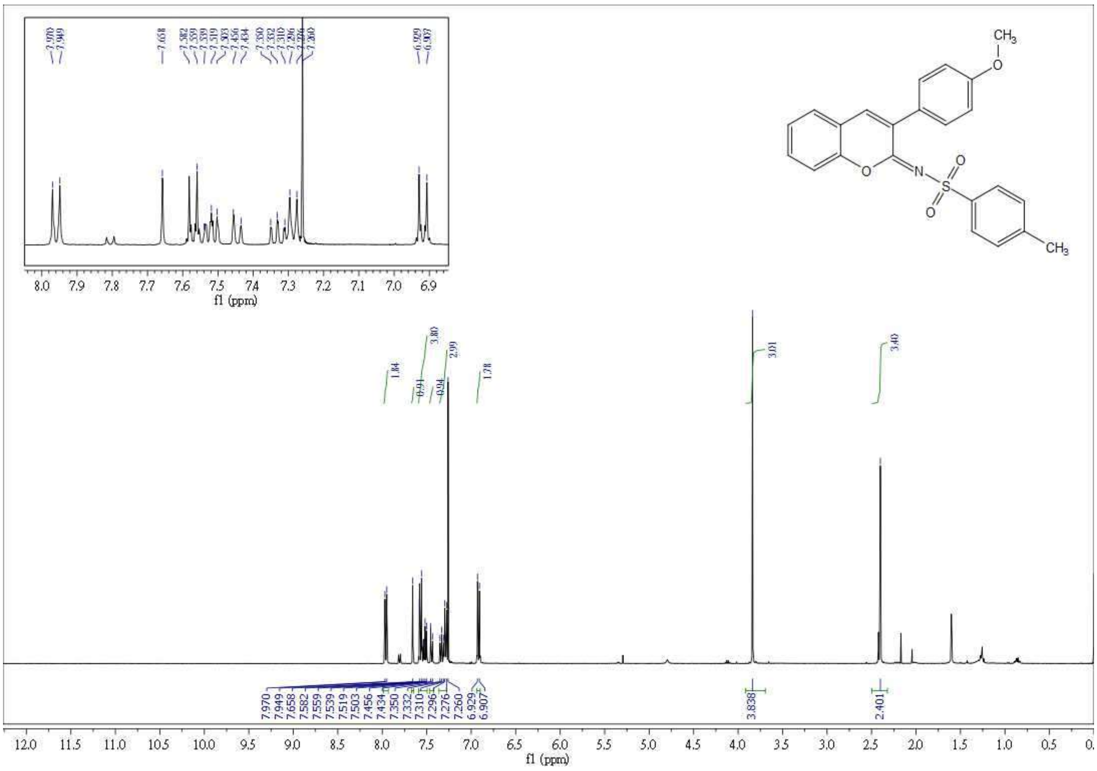

^{13}C NMR spectrum of compound **4j**

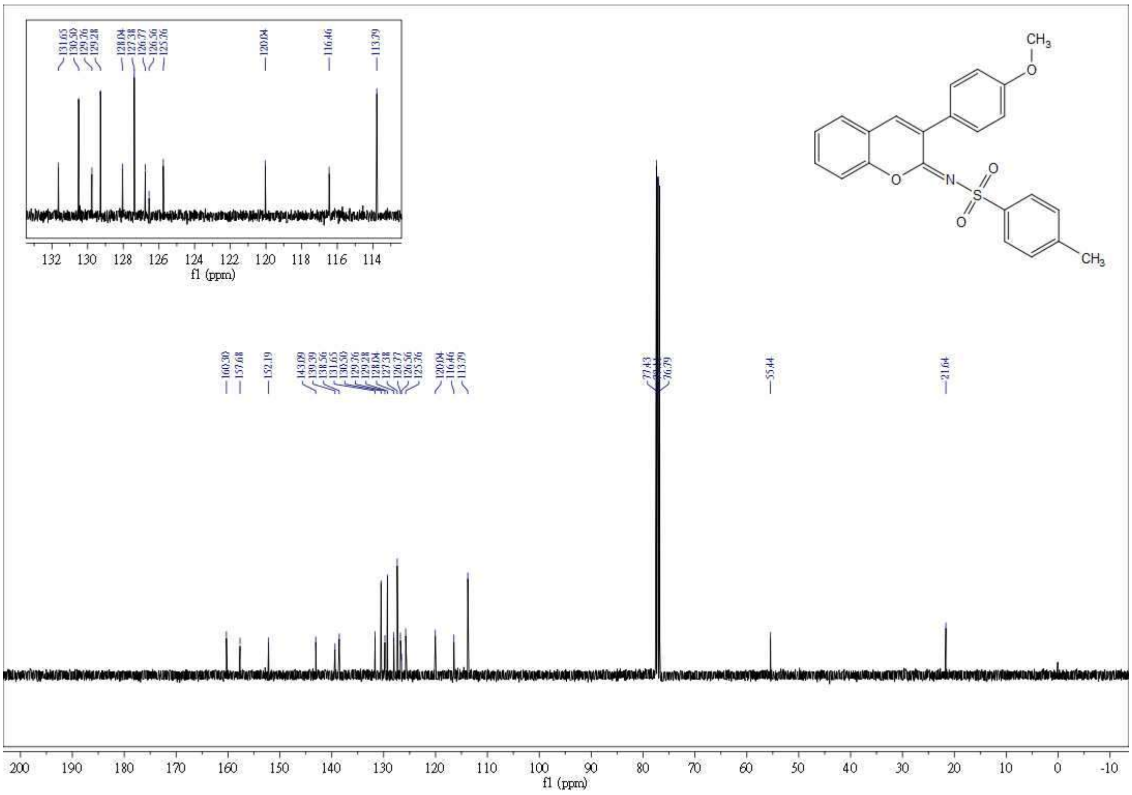

^{1}H NMR spectrum of compound **4k**

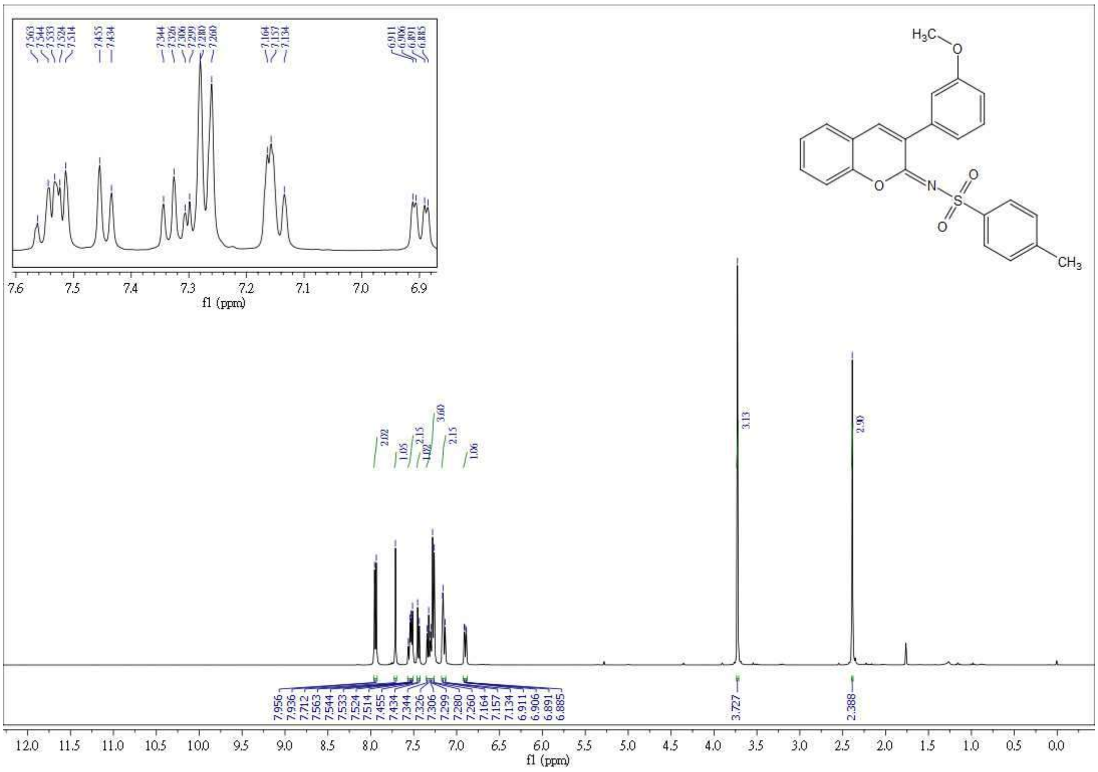

^{13}C NMR spectrum of compound **4k**

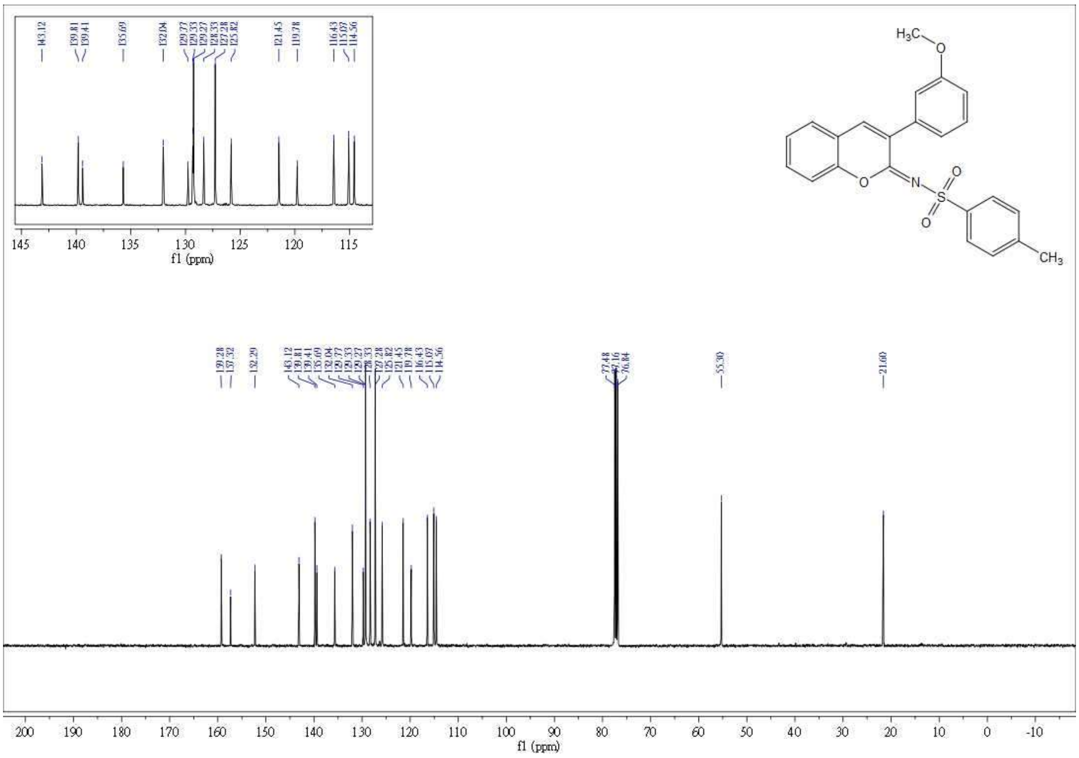

^{1}H NMR spectrum of compound **4l**

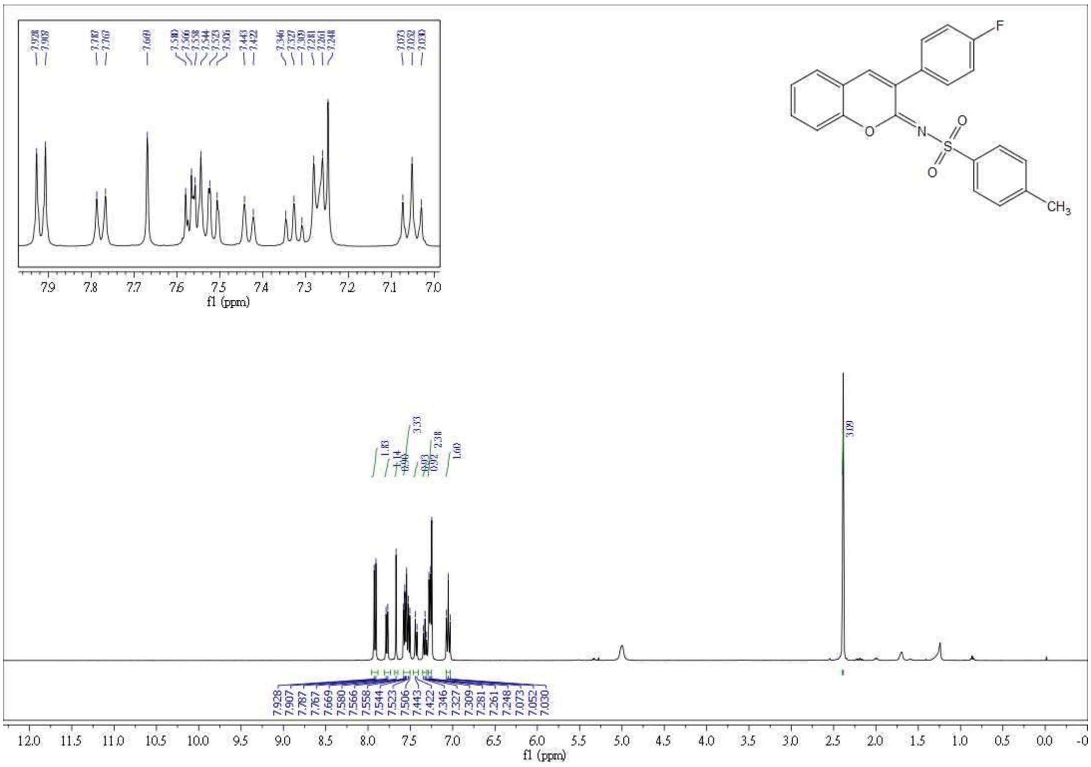

^{13}C NMR spectrum of compound **4l**

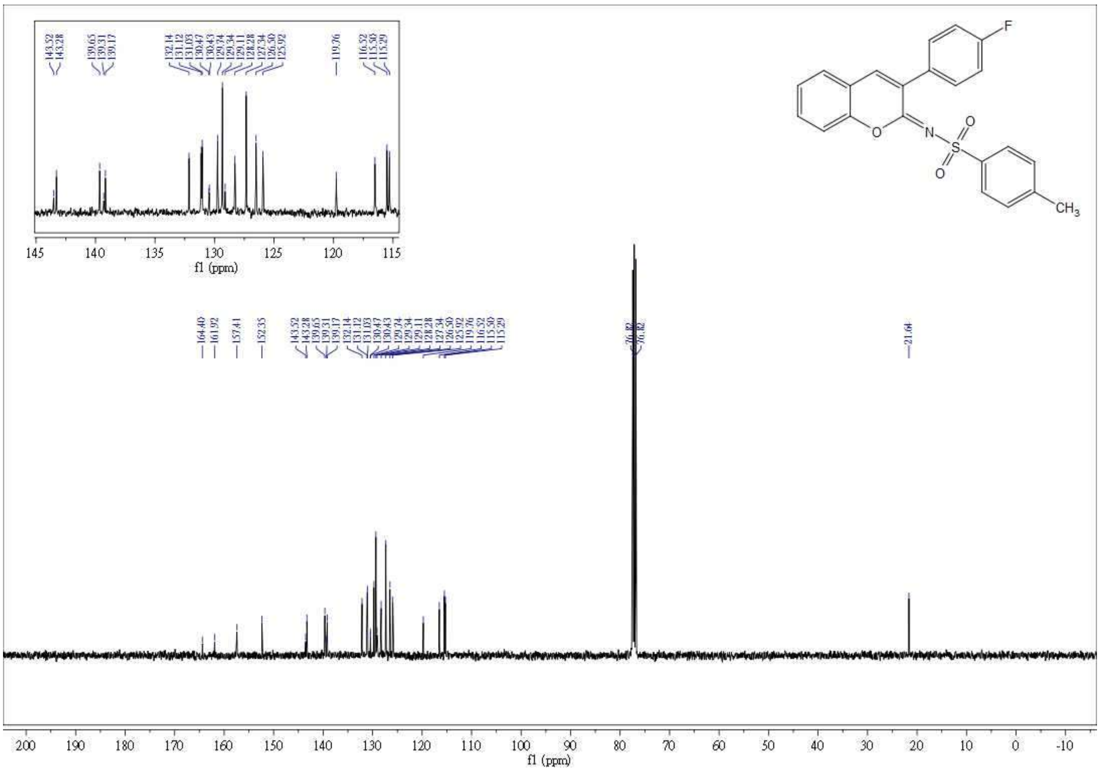

^1H NMR spectrum of compound **4m**

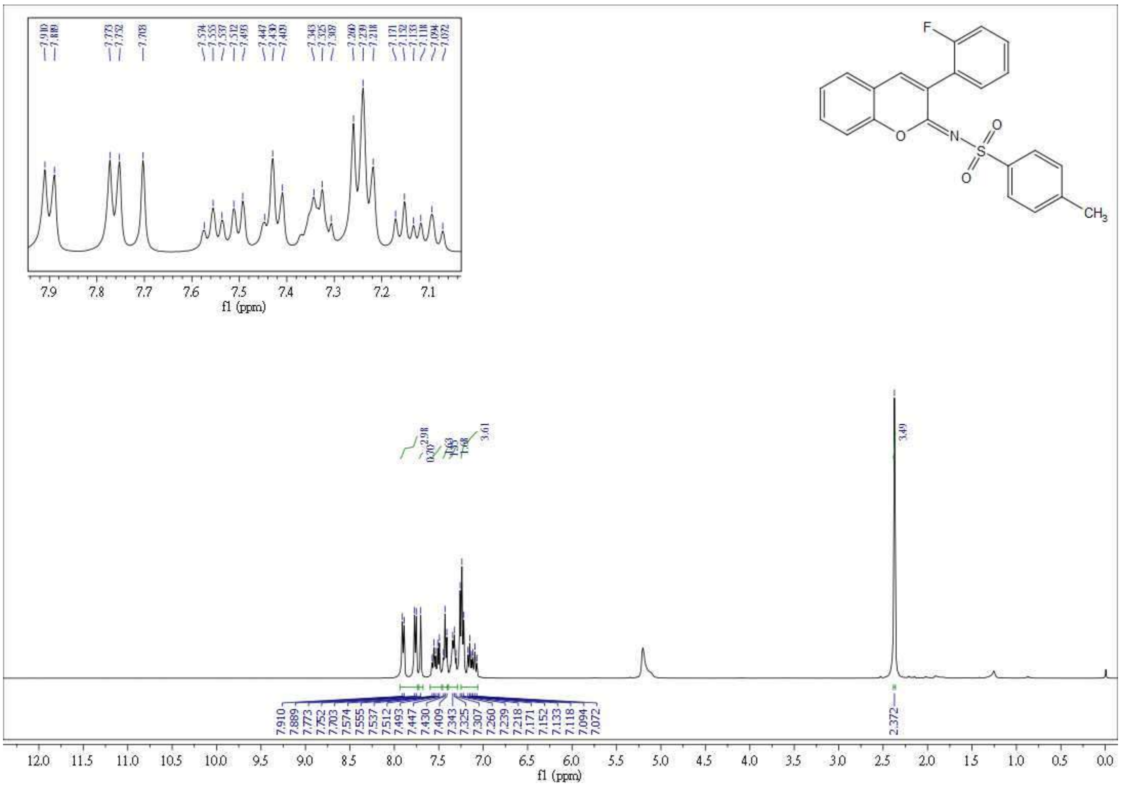

^{13}C NMR spectrum of compound **4m**

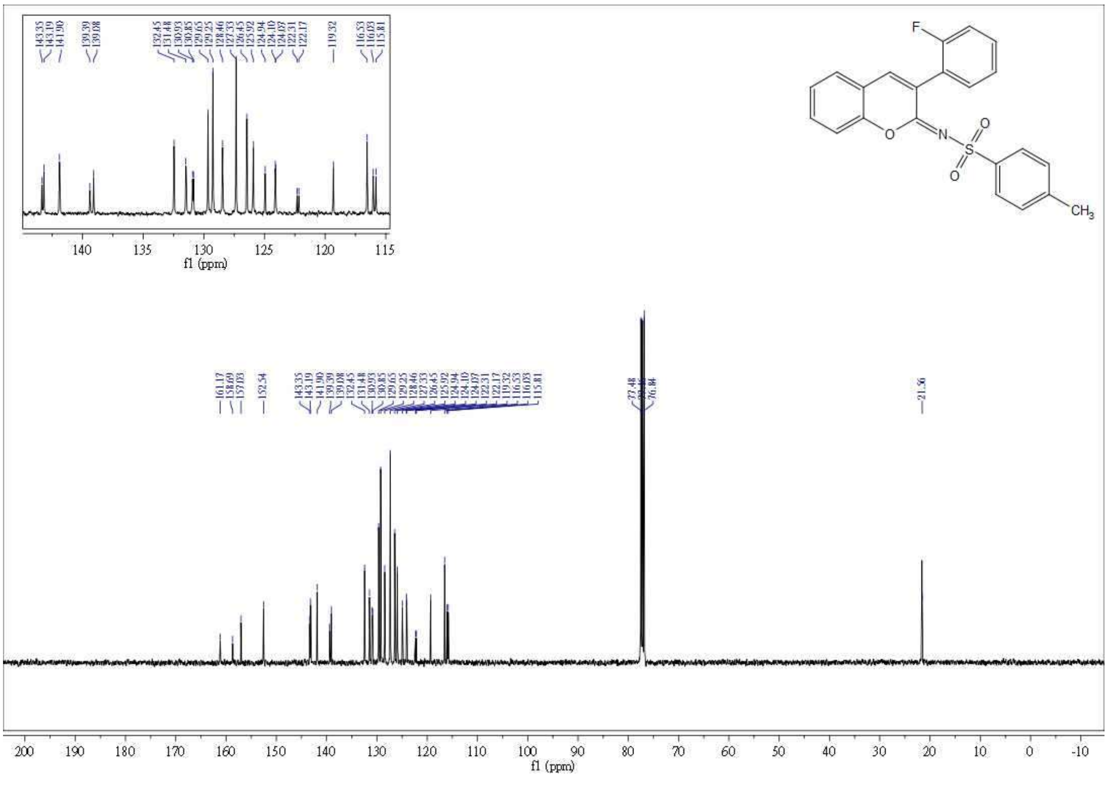

^{1}H NMR spectrum of compound **4n**

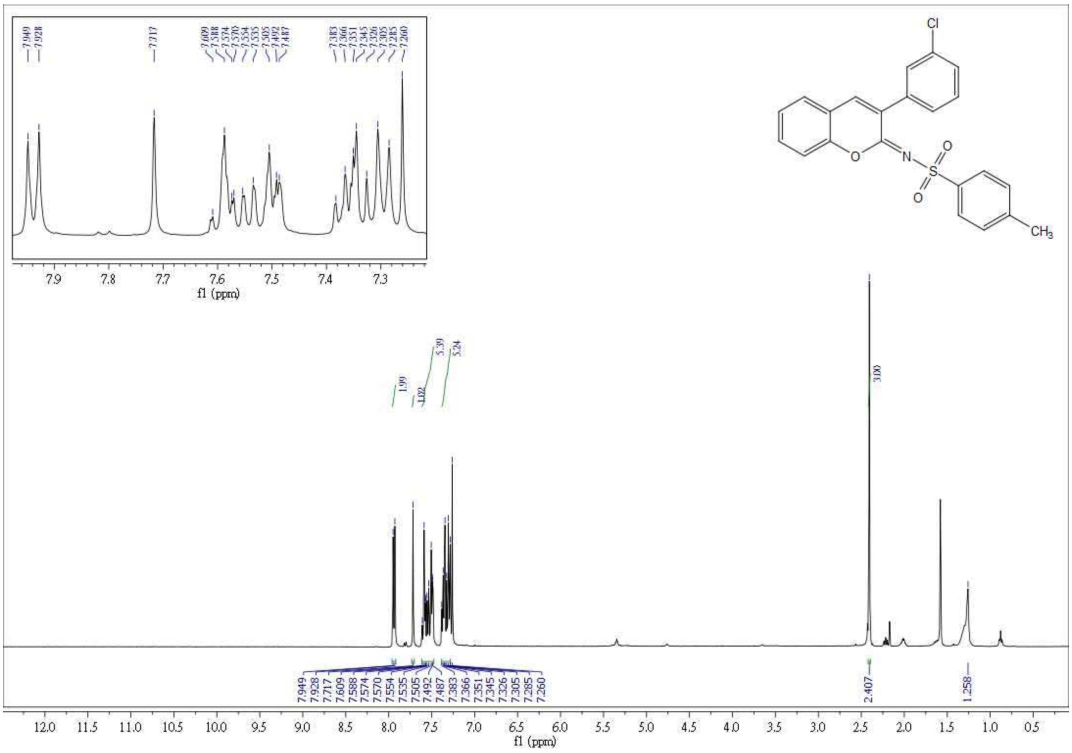

^{13}C NMR spectrum of compound **4n**

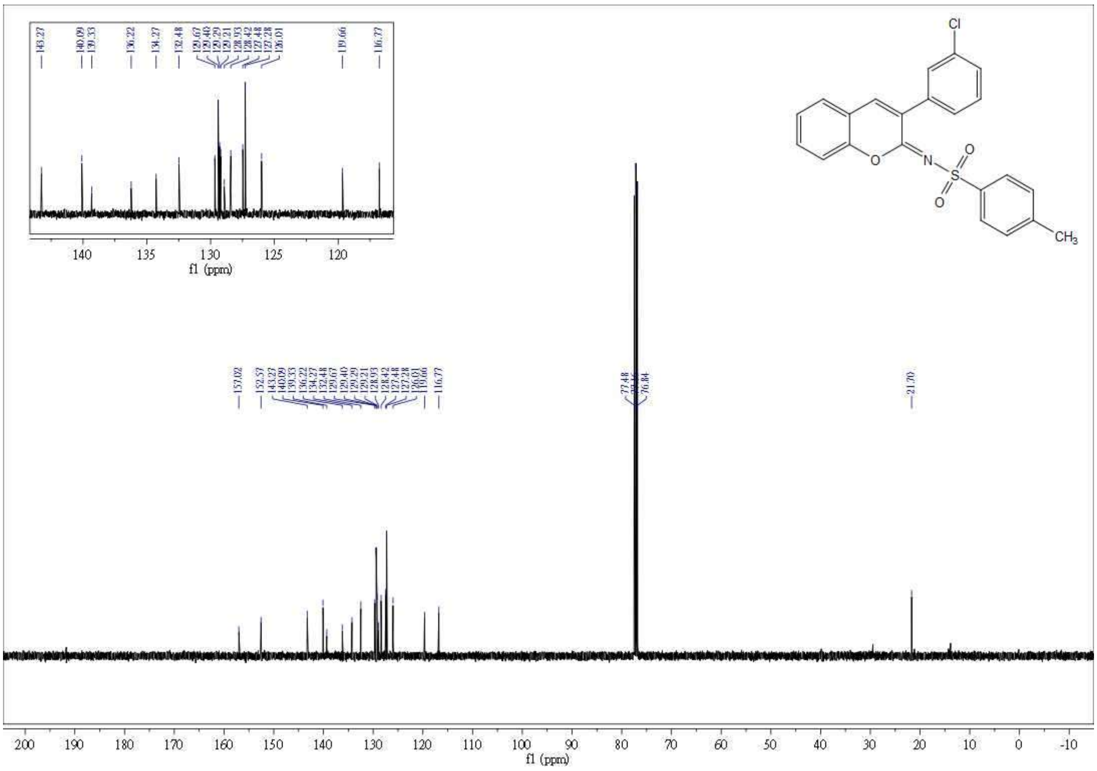

^{1}H NMR spectrum of compound **4o**

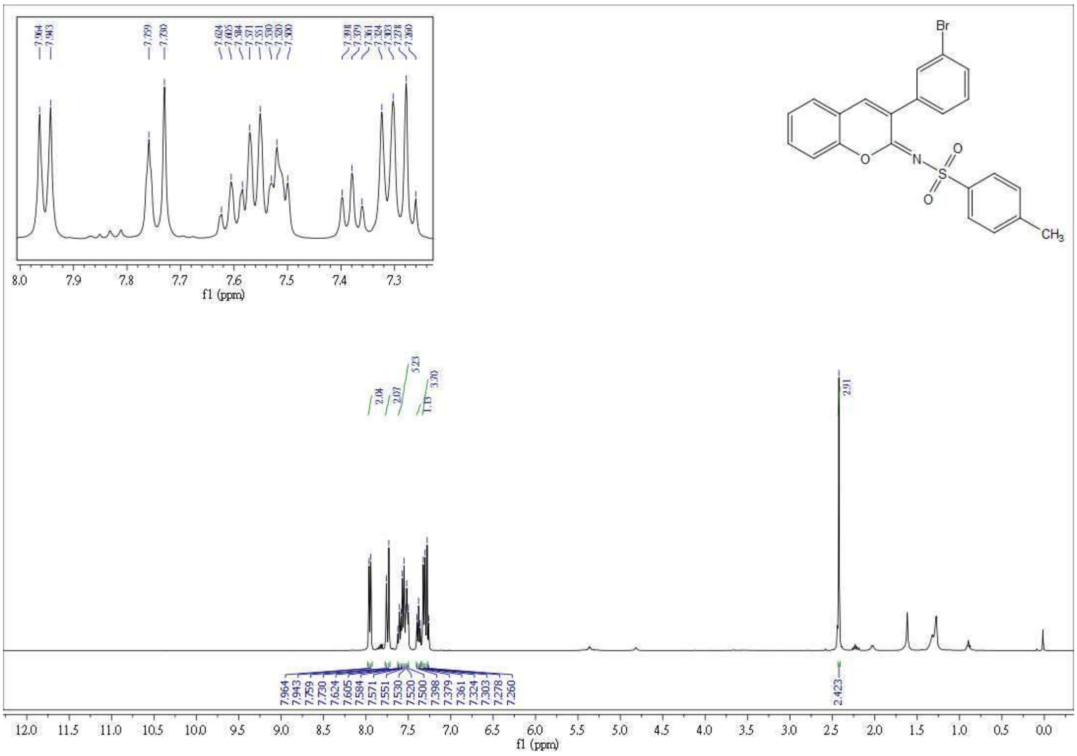

^{13}C NMR spectrum of compound **4o**

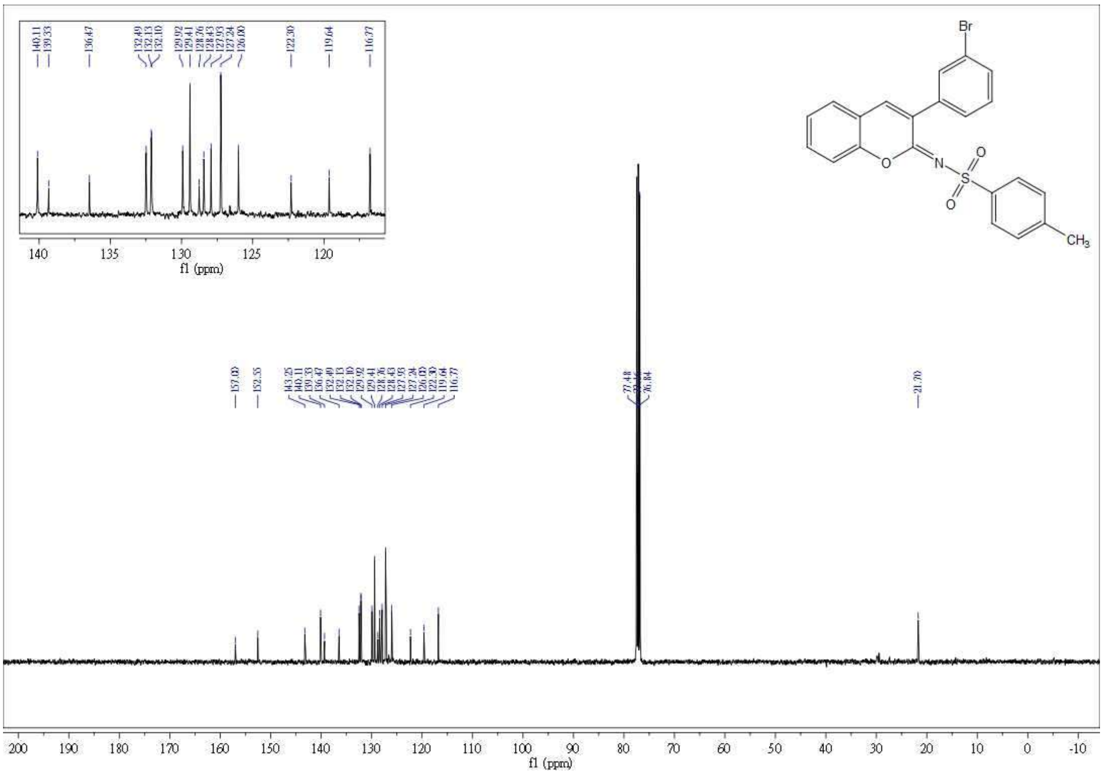

^{1}H NMR spectrum of compound **4p**

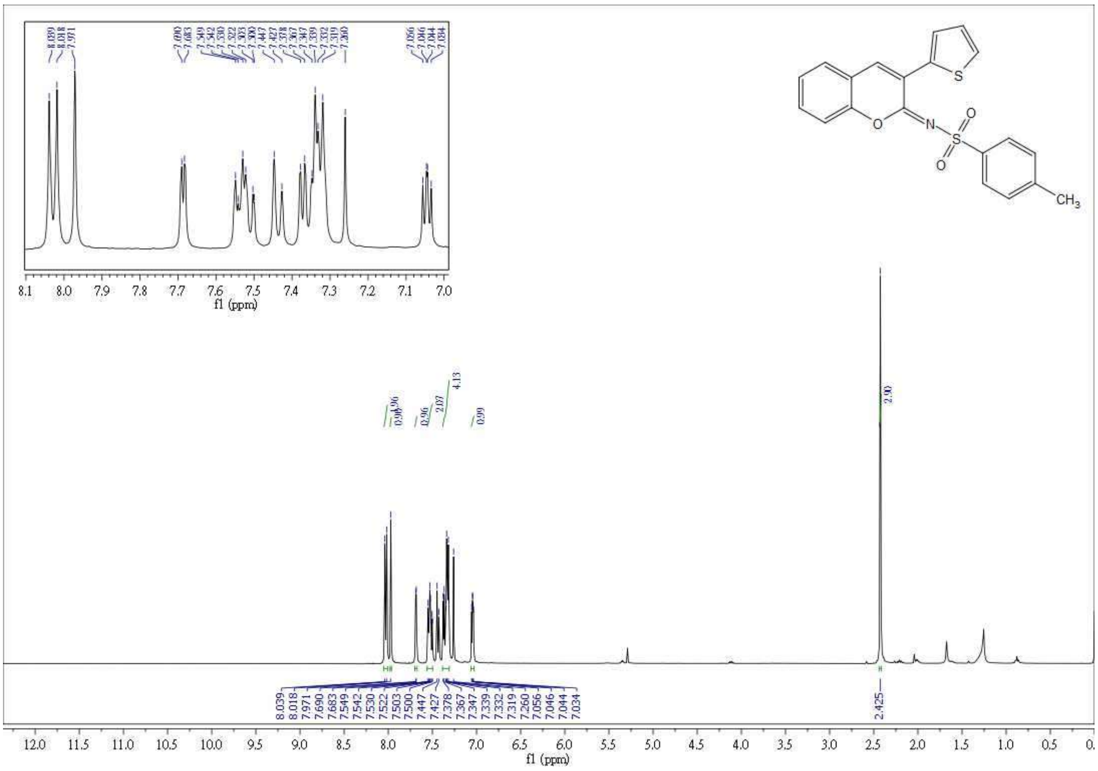

^{13}C NMR spectrum of compound **4p**

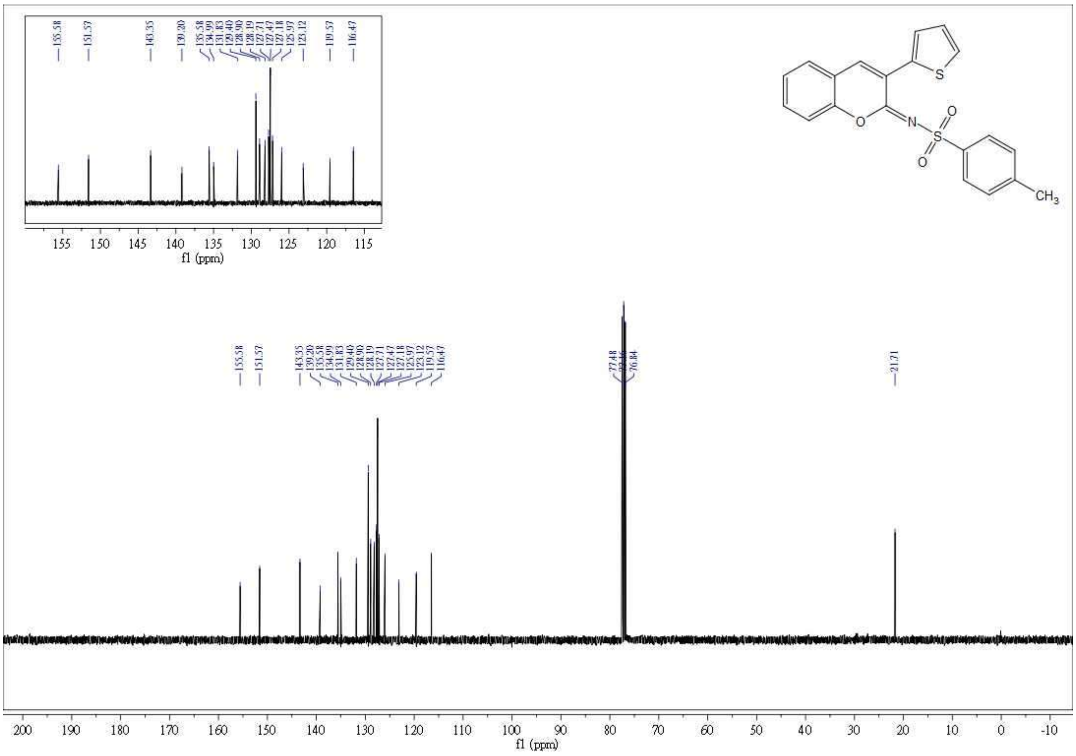

^{1}H NMR spectrum of compound **4q**

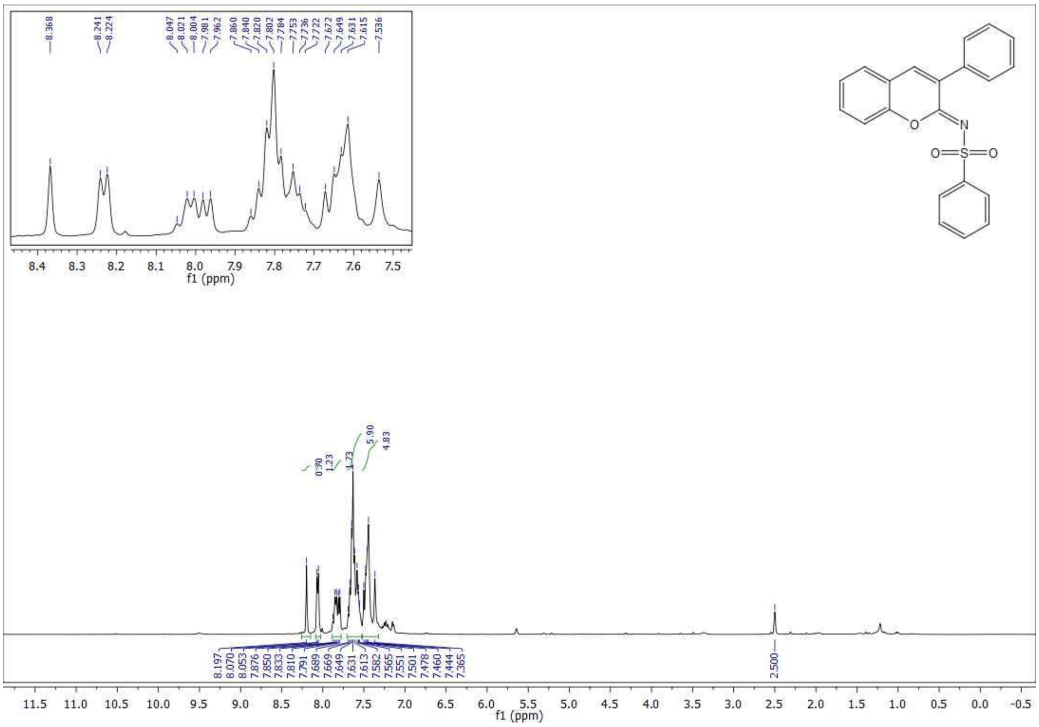

^{13}C NMR spectrum of compound **4q**

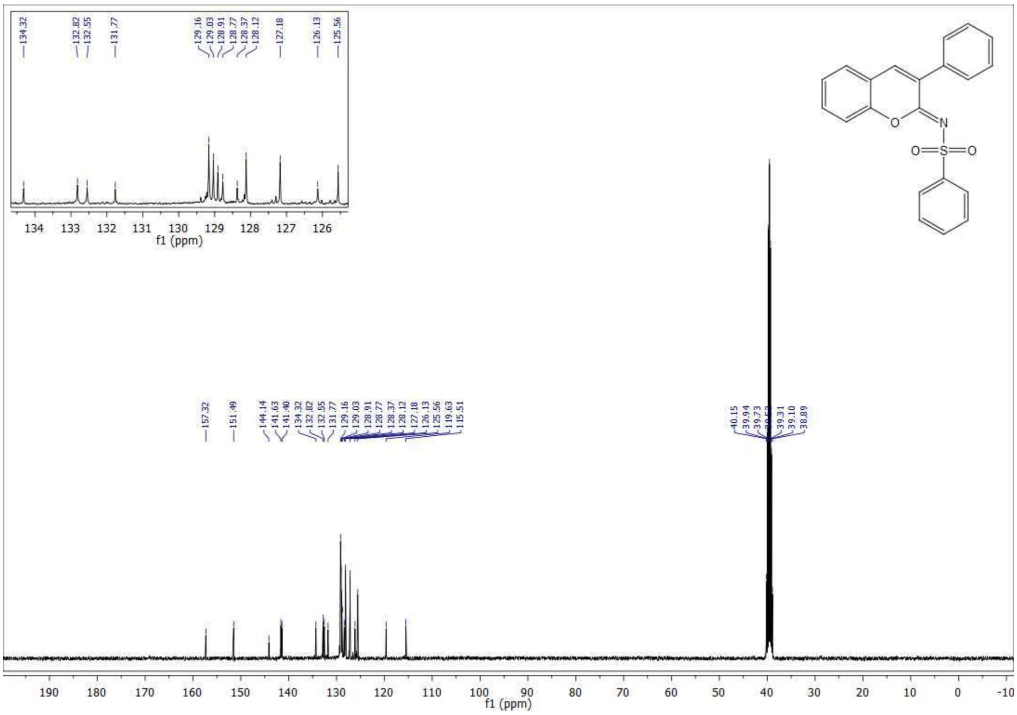

2. X-ray structural details of 4a

Crystal Structure of **4a**

The low temperature (173±2°K) single-crystal X-ray experiments were performed on a SuperNova diffractometer with Cu K_α radiation. Unit cell was obtained and refined by 2964 reflections with 4.2° < θ < 75.6°. No decay was observed in data collection. Raw intensities were corrected for Lorentz and polarization effects, and for absorption by empirical method. Direct phase determination yielded the positions of all non-hydrogen atoms. All non-hydrogen atoms were subjected to anisotropic refinement. All hydrogen atoms were generated geometrically with C-H bonds of 0.93-0.96 Å according to criteria described in the SHELXTL manual (Bruker, 1997). They were included in the refinement with $U_{iso}(H) = 1.2U_{eq}$ or $1.5U_{eq}$ (for methyl C) of their parent atoms. The final full-matric least-square refinement on F^2 converged with $R1 = 0.0385$ and $wR2 = 0.1001$ for 3067 observed reflections [I ≥ 2σ(I)]. The final difference electron density map shows no features. Details of crystal parameters, data collection and structure refinement are given in Table 1.

Data collection was controlled by CrysAlisPro, Agilent Technologies, Version 1.171.36.32 (Oxford, 2013). Computations were performed using the SHELXTL NT ver. 5.10 program package (Bruker, 1997) on an IBM PC 586 computer. Analytic expressions of atomic scattering factors were employed, and anomalous dispersion corrections were incorporated (*International Tables for X-ray Crystallography*, 1989). Crystal drawings were produced with XP (Bruker, 1997).

References

31. Bruker. (1997) SHELXTL. Structure Determination Programs, Version 5.10, Bruker AXS Inc.,6300 Enterprise Lane, Madison, WI 53719-1173, USA.

32. *International Tables for X-ray Crystallography*: (1989) Vol. C (Kluwer Academic Publishers, Dordrecht) Tables 4.2.6.8 and 6.1.1.4.

33. Oxford. (2013) CrysAlisPro, Agilent Technologies, Version 1.171.36.32, Oxford Diffraction Ltd., 68 Milton Park, Abingdon, Oxfordshire, OX14 4RX, UK.

Table 1. Details of Data Collection, Processing and Structure Refinement

Sample code	**4a**
Molecular formula	$C_{22}H_{17}NO_3S$
Molecular weight	375.42
Color and habit	colorless plate
Crystal size	0.02 × 0.12 × 0.20 mm
Crystal system	triclinic
Space group	$P\bar{1}$ (No. 2)
Unit cell parameters	a = 9.5245(4) Å α = 89.450(4)°
	b = 9.9095(6) Å β = 75.509(4)°
	c = 10.9175(5) Å γ = 63.867(5)°
	V = 889.64(9) Å^3 Z = 2 F (000) = 392
Density (calcd)	1.401 g/cm^3
Diffractometer	SuperNova, Dual, Cu at home/near, AtlasS2
Radiation	Cu K_α, λ = 1.54178 Å
Temperature	173±2K
Scan type	ω-scan
Data collection range	$-9 < h < 11$, $-12 < k < 12$, $-10 < l < 13$; $\theta_{max} = 76.0°$
Reflections measured	Total: 5954 Unique (n): 3563 Observed [I ≥ 2σ(I)]: 3067
Absorption coefficient	1.808 mm^{-1}
Minimum and maximum transmission	0.934, 1.000
No. of variables, p	245
Weighting scheme	$w = \frac{1}{\sigma^2(F_o^2)+(0.049P)^2+0.20P}$ $P = (F_o^2 + 2F_c^2)/3$
$R1 = \frac{\Sigma\|\|F_o\|-\|F_c\|\|}{\Sigma\|F_o\|}$ (for all reflections)	0.0460 0.0385 (for observed data)
$wR2 = \sqrt{\frac{\Sigma[w(F_o^2-F_c^2)^2]}{\Sigma w(F_o^2)^2}}$ (for all reflections)	0.1065 0.1001 (for observed data)
Goof = S = $\sqrt{\frac{\Sigma[w(F_o^2-F_c^2)^2]}{n-p}}$	1.031
Largest and mean Δ/σ	0.000, 0.000
Residual extrema in final difference map	-0.384 to 0.232 e Å^{-3}

Table 2. Atomic coordinates and equivalent isotropic temperature factors* (Å^2)

Atoms	x	y	z	$U_{eq.}$
S(1)	0.28536(5)	0.72249(4)	0.21017(4)	0.02437(12)
O(1)	0.20720(15)	0.73905(15)	0.11019(12)	0.0318(3)
O(2)	0.19777(15)	0.83159(14)	0.32196(12)	0.0348(3)
O(3)	0.09486(14)	0.57185(14)	0.29194(11)	0.0268(3)
N(1)	0.35192(17)	0.54997(16)	0.24937(13)	0.0255(3)
C(1)	0.4712(2)	0.72820(19)	0.14391(15)	0.0249(3)
C(2)	0.6071(2)	0.6042(2)	0.07067(16)	0.0273(4)
C(3)	0.7455(2)	0.6197(2)	0.01096(16)	0.0298(4)
C(4)	0.7506(2)	0.7564(2)	0.02348(18)	0.0328(4)
C(5)	0.6130(3)	0.8785(2)	0.0977(2)	0.0439(5)
C(6)	0.4734(2)	0.8653(2)	0.1579(2)	0.0380(4)
C(7)	0.9015(3)	0.7730(3)	-0.0388(2)	0.0443(5)
C(8)	0.25486(19)	0.48952(19)	0.28469(15)	0.0236(3)
C(9)	0.3125(2)	0.33531(19)	0.31800(15)	0.0250(3)
C(10)	0.2020(2)	0.2828(2)	0.36091(16)	0.0278(4)
C(11)	0.0328(2)	0.3707(2)	0.36910(15)	0.0273(4)
C(12)	-0.0849(2)	0.3185(2)	0.40836(18)	0.0353(4)
C(13)	-0.2424(2)	0.4074(3)	0.40435(18)	0.0393(5)
C(14)	-0.2857(2)	0.5490(3)	0.36151(18)	0.0387(5)
C(15)	-0.1733(2)	0.6046(2)	0.32379(17)	0.0331(4)
C(16)	-0.0160(2)	0.5144(2)	0.32923(15)	0.0265(3)
C(17)	0.4885(2)	0.24006(19)	0.30404(16)	0.0260(3)
C(18)	0.6058(2)	0.23034(19)	0.19308(17)	0.0276(4)
C(19)	0.7686(2)	0.1338(2)	0.17823(19)	0.0337(4)
C(20)	0.8179(2)	0.0468(2)	0.2731(2)	0.0404(5)
C(21)	0.7026(2)	0.0564(2)	0.3838(2)	0.0427(5)
C(22)	0.5386(2)	0.1514(2)	0.39871(18)	0.0339(4)

*$U_{eq.}$ defined as one third of the trace of the orthogonalized **U** tensor.

Table 3. Bond lengths (Å) and bond angles (º)

S(1)-O(1)	1.4381(13)	C(9)-C(10)	1.353(2)
S(1)-O(2)	1.4407(13)	C(9)-C(17)	1.484(2)
S(1)-N(1)	1.6354(14)	C(10)-C(11)	1.434(2)
S(1)-C(1)	1.7622(16)	C(11)-C(16)	1.392(3)
O(3)-C(8)	1.3564(19)	C(11)-C(12)	1.405(2)
O(3)-C(16)	1.384(2)	C(12)-C(13)	1.378(3)
N(1)-C(8)	1.291(2)	C(13)-C(14)	1.390(3)
C(1)-C(6)	1.378(2)	C(14)-C(15)	1.383(3)
C(1)-C(2)	1.387(2)	C(15)-C(16)	1.382(2)
C(2)-C(3)	1.391(2)	C(17)-C(22)	1.388(2)
C(3)-C(4)	1.386(3)	C(17)-C(18)	1.399(2)
C(4)-C(5)	1.389(3)	C(18)-C(19)	1.384(2)
C(4)-C(7)	1.508(2)	C(19)-C(20)	1.381(3)
C(5)-C(6)	1.390(3)	C(20)-C(21)	1.386(3)
C(8)-C(9)	1.456(2)	C(21)-C(22)	1.391(3)
O(1)-S(1)-O(2)	117.52(8)	C(10)-C(9)-C(17)	121.78(15)
O(1)-S(1)-N(1)	111.12(8)	C(8)-C(9)-C(17)	120.04(14)
O(2)-S(1)-N(1)	110.79(8)	C(9)-C(10)-C(11)	122.46(16)
O(1)-S(1)-C(1)	107.43(8)	C(16)-C(11)-C(12)	118.01(17)
O(2)-S(1)-C(1)	108.17(8)	C(16)-C(11)-C(10)	117.60(15)
N(1)-S(1)-C(1)	100.28(8)	C(12)-C(11)-C(10)	124.31(17)
C(8)-O(3)-C(16)	122.41(13)	C(13)-C(12)-C(11)	120.08(19)
C(8)-N(1)-S(1)	120.93(12)	C(12)-C(13)-C(14)	120.19(17)
C(6)-C(1)-C(2)	120.40(16)	C(15)-C(14)-C(13)	121.18(18)
C(6)-C(1)-S(1)	117.41(14)	C(16)-C(15)-C(14)	117.86(19)
C(2)-C(1)-S(1)	121.95(13)	C(15)-C(16)-O(3)	117.22(16)
C(1)-C(2)-C(3)	119.31(16)	C(15)-C(16)-C(11)	122.67(16)
C(4)-C(3)-C(2)	121.31(17)	O(3)-C(16)-C(11)	120.11(15)
C(3)-C(4)-C(5)	118.15(17)	C(22)-C(17)-C(18)	118.71(16)
C(3)-C(4)-C(7)	121.53(19)	C(22)-C(17)-C(9)	120.04(15)
C(5)-C(4)-C(7)	120.31(18)	C(18)-C(17)-C(9)	121.13(15)
C(4)-C(5)-C(6)	121.36(18)	C(19)-C(18)-C(17)	120.44(17)
C(1)-C(6)-C(5)	119.46(18)	C(20)-C(19)-C(18)	120.61(17)
N(1)-C(8)-O(3)	119.32(15)	C(19)-C(20)-C(21)	119.36(17)
N(1)-C(8)-C(9)	121.65(15)	C(20)-C(21)-C(22)	120.43(18)
O(3)-C(8)-C(9)	119.03(14)	C(17)-C(22)-C(21)	120.44(17)
C(10)-C(9)-C(8)	118.18(15)		

Table 4. Anisotropic thermal parameters* (Å^2)

Atoms	U_{11}	U_{22}	U_{33}	U_{23}	U_{13}	U_{12}
S(1)	0.0213(2)	0.0214(2)	0.0276(2)	0.00408(15)	-0.00585(15)	-0.00762(16)
O(1)	0.0268(6)	0.0344(7)	0.0371(7)	0.0124(5)	-0.0143(5)	-0.0137(5)
O(2)	0.0328(7)	0.0281(7)	0.0336(7)	-0.0027(5)	-0.0003(5)	-0.0095(6)
O(3)	0.0202(6)	0.0288(6)	0.0299(6)	0.0056(5)	-0.0061(5)	-0.0102(5)
N(1)	0.0228(7)	0.0238(7)	0.0293(7)	0.0056(6)	-0.0065(6)	-0.0104(6)
C(1)	0.0247(8)	0.0262(8)	0.0256(8)	0.0042(6)	-0.0078(6)	-0.0126(7)
C(2)	0.0283(8)	0.0255(8)	0.0287(8)	0.0007(7)	-0.0067(7)	-0.0132(7)
C(3)	0.0261(8)	0.0346(10)	0.0278(9)	0.0017(7)	-0.0064(7)	-0.0134(7)
C(4)	0.0326(9)	0.0413(10)	0.0334(9)	0.0085(8)	-0.0125(7)	-0.0226(8)
C(5)	0.0418(11)	0.0325(10)	0.0643(14)	0.0034(9)	-0.0107(10)	-0.0249(9)
C(6)	0.0335(10)	0.0248(9)	0.0516(12)	-0.0023(8)	-0.0048(9)	-0.0132(8)
C(7)	0.0404(11)	0.0580(13)	0.0458(12)	0.0075(10)	-0.0096(9)	-0.0335(11)
C(8)	0.0216(8)	0.0262(8)	0.0214(7)	0.0028(6)	-0.0059(6)	-0.0093(7)
C(9)	0.0247(8)	0.0265(8)	0.0231(8)	0.0044(6)	-0.0056(6)	-0.0114(7)
C(10)	0.0304(9)	0.0295(9)	0.0253(8)	0.0072(7)	-0.0074(7)	-0.0154(7)
C(11)	0.0272(8)	0.0364(9)	0.0214(8)	0.0035(7)	-0.0063(6)	-0.0172(8)
C(12)	0.0361(10)	0.0471(11)	0.0314(9)	0.0069(8)	-0.0076(8)	-0.0274(9)
C(13)	0.0324(10)	0.0619(13)	0.0330(10)	0.0022(9)	-0.0069(8)	-0.0306(10)
C(14)	0.0253(9)	0.0604(13)	0.0312(9)	0.0014(9)	-0.0101(7)	-0.0187(9)
C(15)	0.0258(9)	0.0431(11)	0.0277(9)	0.0037(8)	-0.0088(7)	-0.0127(8)
C(16)	0.0237(8)	0.0370(9)	0.0197(8)	0.0018(7)	-0.0045(6)	-0.0153(7)
C(17)	0.0241(8)	0.0229(8)	0.0321(9)	0.0057(7)	-0.0084(7)	-0.0112(7)
C(18)	0.0266(8)	0.0248(8)	0.0320(9)	0.0049(7)	-0.0069(7)	-0.0127(7)
C(19)	0.0248(8)	0.0284(9)	0.0456(11)	0.0017(8)	-0.0040(8)	-0.0128(7)
C(20)	0.0235(9)	0.0293(10)	0.0646(14)	0.0075(9)	-0.0137(9)	-0.0078(8)
C(21)	0.0359(10)	0.0356(11)	0.0549(13)	0.0206(9)	-0.0192(9)	-0.0115(9)
C(22)	0.0308(9)	0.0329(10)	0.0364(10)	0.0114(8)	-0.0081(7)	-0.0139(8)

*The exponent takes the form: $-2\pi^2\Sigma\Sigma U_{ij}h_ih_j\mathbf{a}_i^*\mathbf{a}_j^*$

Table 5. Coordinates and isotropic temperature factors* (Å^2) for H atoms

Atoms	x	y	z	$U_{eq.}$
H(2)	0.6056	0.5116	0.0616	0.033
H(3)	0.8366	0.5367	-0.0384	0.036
H(5)	0.6144	0.9710	0.1074	0.053
H(6)	0.3823	0.9482	0.2071	0.046
H(7A)	0.9509	0.7826	0.0252	0.066
H(7B)	0.8730	0.8616	-0.0827	0.066
H(7C)	0.9769	0.6854	-0.0985	0.066
H(10)	0.2371	0.1860	0.3860	0.033
H(12)	-0.0563	0.2239	0.4371	0.042
H(13)	-0.3201	0.3726	0.4304	0.047
H(14)	-0.3921	0.6074	0.3581	0.046
H(15)	-0.2026	0.6997	0.2957	0.040
H(18)	0.5743	0.2891	0.1288	0.033
H(19)	0.8455	0.1275	0.1037	0.040
H(20)	0.9275	-0.0176	0.2629	0.048
H(21)	0.7352	-0.0010	0.4484	0.051
H(22)	0.4619	0.1555	0.4726	0.041

*The exponent takes the form: $-8\pi^2 U \sin^2\theta/\lambda^2$

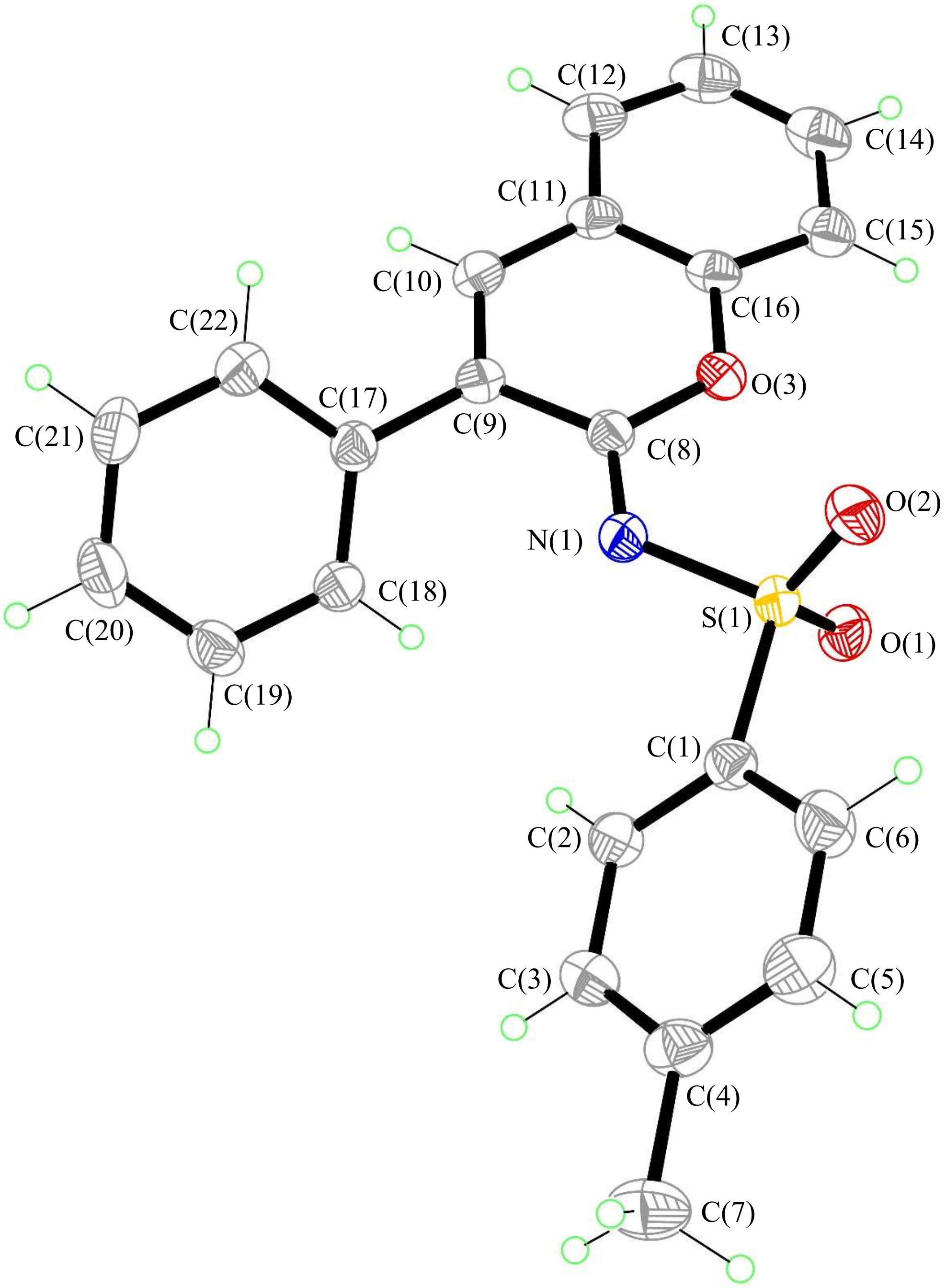

ORTEP drawing of $C_{22}H_{17}NO_3S$ with 50% probability ellipsoids, showing the atomic numbering scheme.

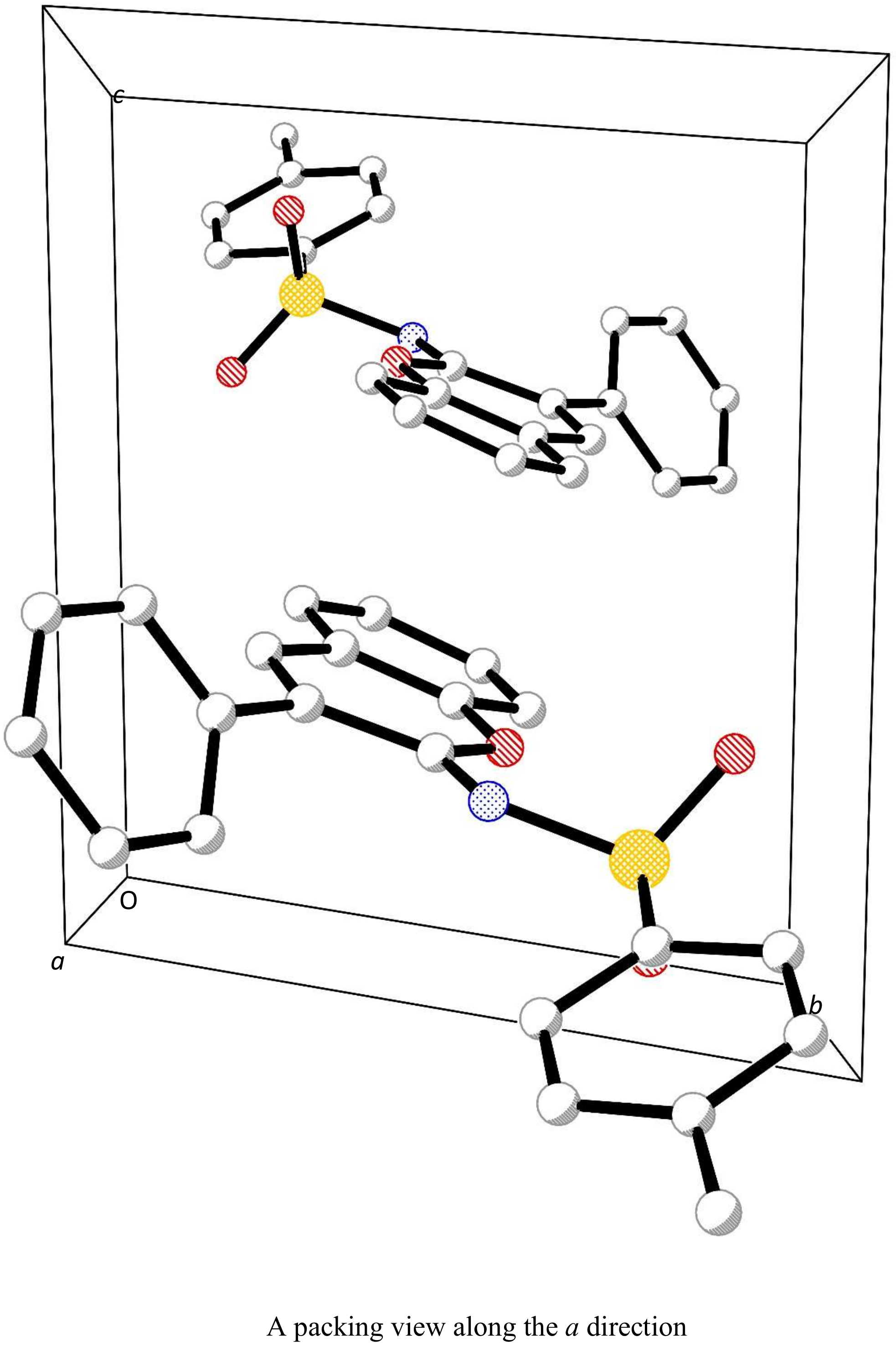

A packing view along the *a* direction
